# PGIP: a web server for the rapid taxonomic identification of parasite genomes

**DOI:** 10.1186/s13071-025-07007-3

**Published:** 2025-08-28

**Authors:** Jiayao Zhang, Feng Tang, Bixian Ni, Qiang Zhang, Xinyi Gong, Fanzhen Mao, Jun Cao, Yaobao Liu

**Affiliations:** 1 National Health Commission Key Laboratory of Parasitic Disease Control and Prevention, Jiangsu Provincial Key Laboratory on Parasite and Vector Control Technology, Jiangsu Provincial Medical Key Laboratory, Jiangsu Institute of Parasitic Diseases, Wuxi, 214064 Jiangsu China; 2 School of Public Health, Nanjing Medical University, Nanjing, 211166 Jiangsu China

**Keywords:** Parasite identification, Parasite genomics, Metagenomics next-generation sequencing, Bioinformatics platform, Web server

## Abstract

**Background:**

Parasitic diseases remain a global health challenge, and traditional methods in their diagnosis face limitations in sensitivity and scalability. Genome-based sequencing technologies have improved and are increasingly employed for the identification of parasites; however, their clinical adoption remains hindered by the complexity of bioinformatics analysis, reliance on incomplete reference databases, and accessibility barriers for nonspecialists. Overcoming these challenges necessitates the development of standardized analytical workflows and high-quality genomic resources specifically tailored for parasite identification.

**Methods:**

We developed a user-friendly web server named the Parasite Genome Identification Platform (PGIP). The reference database was sourced from the National Center for Biotechnology Information (NCBI), WormBase, European Nucleotide Archive (ENA), and VEuPathDB, rigorously filtered for quality, and deduplicated using Cluster Database at High Identity with Tolerance (CD-HIT) to ensure accuracy and nonredundancy. To streamline analysis, we integrated a standardized identification pipeline built on Nextflow, which encompasses host DNA depletion, quality control, parasite species identification via both reads mapping and assembly-based approaches, and automated report generation for comprehensive diagnostic insights.

**Results:**

PGIP integrates a curated database of 280 parasite genomes; which is rigorously filtered for quality and taxonomic accuracy. Validation across diverse datasets demonstrated the precise species-level resolution of PGIP, and its compatibility with clinical samples. The platform features an intuitive graphic interface; and one-click analysis significantly reduces reliance on bioinformatics expertise, thus enabling rapid diagnosis.

**Conclusions:**

PGIP offers an accurate, efficient, and a user-friendly web server designed to simplify and accelerate the taxonomic identification of parasite genomes using data from metagenomic next-generation sequencing. Its automated framework reduces the need for specialized expertise, enabling rapid application in clinical and public health settings.

**Graphical Abstract:**

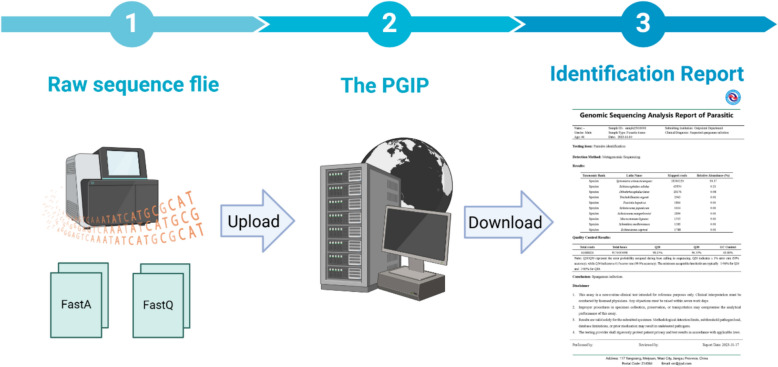

**Supplementary information:**

The online version contains supplementary material available at 10.1186/s13071-025-07007-3.

## Background

Parasitic diseases remain a significant global public health challenge, affecting millions and causing substantial morbidity and mortality worldwide [1–3]. While improved sanitation has reduced prevalence in many regions, this progress has paradoxically increased diagnostic challenges owing to diminished clinical familiarity with rare infections [4]. In this context of better control and lower prevalence, sporadic re-emergences of parasitic diseases occur; in part due to changes in climate and ecological conditions, as well as increased international travel. This phenomenon further complicates disease surveillance efforts and poses challenges to clinical diagnosis [5].

The accurate identification of parasites is essential for a comprehensive disease control strategy. Traditional microscopic analysis requires a high level of parasitological knowledge combined with the ability to objectively assess morphological variations. Microscopy is labor and time intensive, with low sensitivity and reproducibility [6]. For the diagnosis of rare parasites, morphological identification is frequently compromised by suboptimal sample preservation or delays in processing, thus hindering definitive diagnosis.

Metagenomic next-generation sequencing (mNGS) is notable for its unbiased, high-throughput nature, which enables direct sequencing of nucleic acids from diverse clinical samples [7]. By eliminating the requirement for pathogen-specific hypotheses during diagnosis, mNGS facilitates rapid detection of both known and novel pathogens, including bacteria, fungi, and parasites, thereby enhancing clinical diagnostic accuracy [8–11]. This capability has driven its adoption by researchers, public health officials, and clinicians; particularly in diagnosing parasitic diseases and monitoring outbreaks [12, 13].

However, the clinical adoption of mNGS faces critical barriers, including bioinformatics complexity requiring command-line proficiency, incomplete or taxonomically biased reference databases limiting identification accuracy, and a lack of standardized workflows for parasite identification [14, 15].

To address the challenges in sequencing data analysis faced by professionals who lack bioinformatics expertise, we developed the Parasite Genome Identification Platform (PGIP); a cloud-based bioinformatics platform with a user-friendly graphical interface and standardized automated analysis workflows. This platform facilitates the rapid analysis of sequencing data. Furthermore, to resolve inconsistencies in parasite reference genome databases observed in existing platforms, we developed a specialized, high-accuracy parasite reference genome database to ensure precise identification outcomes.

## Methods

### Database construction and curation

The reference genome data for parasites were sourced from multiple publicly accessible genomic repositories, including the National Center for Biotechnology Information (NCBI) [16], WormBase [17], MalariaGEN [18], the European Nucleotide Archive (ENA) [19], and VEuPathDB [20]. In addition, comprehensive genomic resources were systematically curated through rigorous analysis of peer-reviewed publications that reported whole-genome sequencing assemblies and annotations for parasite species.

Following data retrieval, we implemented rigorous quality control procedures to verify data integrity and consistency, thereby eliminating low-quality or erroneous entries and ensuring overall reliability. For NCBI-annotated reference genomes, we systematically organized genomic metadata into a structured relational database and constructed search indices to enable computationally efficient queries. To construct a high-quality and nonredundant reference database, genome assemblies were screened on the basis of the following criteria: complete genome annotation for coding sequences, and accurate species-level taxonomic classification [21]. Redundant sequences were removed using CD-HIT (v4.8.1) [22] with a sequence identity threshold of 95%. Ambiguous or conflicting taxonomic labels were manually curated through literature review and cross-referencing with the NCBI taxonomy database.

To optimize query efficiency, the database was indexed using memory-mapped technology and structurally optimized to enable rapid large-scale data retrieval. Following its construction, the database was validated with sequencing data from reference samples to ensure accuracy and consistency. Given the dynamic nature of genomic data, the database is scheduled for quarterly updates. These updates will adhere to a standardized protocol involving automated data retrieval pipelines, multistage quality control measures, and peer-reviewed manual curation​ to preserve longitudinal data integrity and clinical relevance.

### Data management

Efficient data management was essential to the development and deployment of PGIP for parasite genome identification. Upon submission, sequencing files (in FASTQ/FASTA format) were securely stored in a distributed file system; and systematically organized by project identifiers, sample metadata, and submission timestamps. This systematic structure streamlined retrieval and preserved data integrity during analysis. To safeguard sensitive data, all transmissions employed protocols such as HTTPS and AES-256 encryption, while role-based access control (RBAC) enforced strict privacy compliance. The platform adhered to a data retention policy under which analysis results were securely stored for 180 days prior to archiving; and accompanied by automated notifications to users before deletion and provisions for long-term export and preservation. This comprehensive data management strategy ensures that PGIP operates with high efficiency, accuracy, and data integrity; while providing reliable and reproducible results for parasite genome identification.

### Design and workflow of PGIP

#### Data preprocessing

The PGIP supports the input of raw paired-end sequencing data in FASTQ format and preprocessed FASTA-formatted sequences from NGS platforms, including its compressed file (such as.gz,.tar). The maximum data size for each sample is 20 Gb. To ensure analytical accuracy, raw FASTQ inputs are subjected to stringent quality control (QC) prior to downstream analysis, including artifact removal and filtration of nontarget sequences. The standardized QC workflow is composed of three critical steps:

1. Adapter removal: sequencing adapters which were introduced during library preparation are systematically trimmed to minimize platform-specific bias using Trimmomatic [23].
2. Quality filtering: low-quality reads (Phred score < 20) and short fragments (< 50 bp) are filtered using Trimmomatic [23]. Quality metrics (e.g., per-base sequence quality, GC content) are visualized using FastQC [24] before and after processing to validate improvements.
3. Host DNA depletion: reads are aligned to the host reference genome (e.g., GRCh38 for human samples) using Bowtie2 v2.4.5 [25] with sensitivity parameters (very-sensitive–local). Nonhost reads (unmapped reads) are retained for downstream pathogen analysis.

#### Parasite identification

Following QC, the cleaned data are automatically analyzed through identification modules within PGIP, which executes taxonomic classification and generates diagnostic reports. These modules utilize two identification methods: the identification of parasite genomes based on reads mapping, and the analysis of assembled data.

#### Reads mapping-based identification of parasite genomes

Kraken2 was used to construct a comprehensive reference genome database for the studied parasites [26]. Genome sequences were indexed using the Kraken2-build command to enable rapid sequence retrieval and alignment. The database was composed of a comprehensive collection of reference genomes for human and zoonotic parasites, such as helminths and protozoa. This taxonomic diversity ensured broad coverage of clinically relevant species, thereby enhancing the accuracy and reliability of parasite identification.

Species identification was performed using a Kraken2 k-mer-based alignment, which classifies sequencing reads against the reference database. Kraken2 segments each sequence into k-mers (contiguous nucleotide subsequences) and matches these to precomputed, taxon-specific k-mers in the database; thereby enabling fast and precise taxonomic classification. The database and its index file were memory-mapped to enable rapid access. Query sequences were split into k-mers and aligned to the reference database to assign taxonomic labels and calculate alignment counts. A hierarchical classification tree was constructed from the taxon-specific alignment scores, with the taxonomic lineage corresponding to the highest cumulative alignment score assigned as the definitive classification. This method also quantified the relative abundance of each parasite species within the sequencing dataset.

The Kraken2 output was composed of species identification results accompanied by detailed taxonomic information. Statistical analyses were performed to generate ecological indices; including species composition, paired read counts, and relative abundance.

#### Assembly-based identification of parasite genomes

The clean sequencing data were assembled using MEGAHIT [27], which constructs extended contig sequences through the iterative assembly of short reads. This assembler employs a multi-k-mer iterative strategy to construct simplified de Bruijn graphs (SdBG) through stepwise optimization cycles (k = 21–141 with 12 bp increments). During iterative assembly, smaller k-mers (21–129 bp) facilitated error correction and gap closure in low-coverage regions by filtering spurious connections and enhancing sequence continuity. Conversely, larger k-mers (141 bp) improved resolution of homologous repetitive elements through extended sequence context analysis [28]. Following each assembly iteration, systematic graph refinement procedures were implemented; including: (1) trimming terminal branches (tips) < 2 kbp, (2) collapsing parallel sequence variants (bubbles) with ≥ 95% similarity, and (3) eliminating graph edges which demonstrated local coverage below 2 × . These optimization strategies collectively generated high-fidelity contigs with enhanced structural integrity and sequence accuracy for downstream analyses.

Taxonomic binning was subsequently performed using MetaBAT [29], a probabilistic clustering tool that integrates contig abundance profiles and tetranucleotide frequency (TNF) patterns to reconstruct metagenome-assembled genomes (MAGs). Leveraging the taxonomically conserved nature of oligonucleotide composition in microbial genomes, MetaBAT first calculated the TNF-based probabilistic distances between contigs, which reflect sequence compositional similarity. Simultaneously, abundance profiles were derived from read alignment depths across samples, to capture genomic coverage variations indicative of population-specific replication rates. These two metrics were empirically weighted to construct a composite probabilistic distance matrix, which enables iterative hierarchical clustering of contigs through a graph-based algorithm. The resulting bins exhibited high phylogenetic resolution, with minimal cross-clade contamination, as validated by marker gene completeness and redundancy assessments.

Finally, taxonomic classification of MAGs was performed using the Contig Annotation Tool (CAT, v5.2) [30]. The CAT function classified long DNA sequences and MAGs by performing gene prediction, aligning open reading frames (ORFs) to the NR protein database, and the usage of a majority voting mechanism for taxonomic assignment based on individual ORFs. The resulting classification scores were analyzed to identify parasite species within the bins.

### Integration of workflow and report output

Integrated analytical workflows were developed using Nextflow [31] to systematically execute multiple bioinformatics processes. To generate an identification report, a Python program was used to extract the 10 most identified parasites from the results; including the Latin names of the detected parasites, the number of detected sequences, and their relative abundance (Relative abundance = species-specific reads ×100/ total reads identified at the species level). The identification report also includes the data quality control results.

### Evaluation of parasite identification

To evaluate the performance of PGIP, we selected a panel of public databases and in-house sequencing datasets representing clinically relevant human parasites. Parasite species were selected to ensure taxonomic diversity and include soil-transmitted helminths (e.g., *Ascaris lumbricoides*), food-borne parasites (e.g., *Clonorchis sinensis*), vector-borne parasites (e.g., *Plasmodium* spp.), and morphologically similar species from the same genus (e.g., *Schistosoma japonicum* and *Schistosoma haematobium*). We utilized sequencing data from diverse specimen types to assess platform performance under varying levels of host-derived contamination. These included stool sample (characterized by substantial background interference from host and microbial sources), blood sample (containing abundant host background), cerebrospinal fluid sample (with limited host content), parasitic sample (exhibiting minimal host interference), and amplicon sequencing sample (PCR-amplified parasitic gene fragments). Furthermore, a negative sample was included in the evaluation.

Public datasets were obtained from the European Nucleotide Archive (ENA). The remaining sequencing datasets were generated in-house as part of the parasitic disease surveillance project conducted at the Jiangsu Institute of Parasitic Diseases (for details, see Supplementary File S2: Datasets for evaluation of parasite identification).

The sequencing data were uploaded to the platform, and the default analysis workflow (read-mapping-based identification module) was executed. This approach directly maps high-quality sequencing reads to the curated parasite genome database, and is optimized for clinical and metagenomic samples without requiring genome assembly. The assembly-based mode is also available within the platform for users who wish to analyze preassembled contigs or scaffolds.

## Results

### Parasite reference genomes database

The final database contained 280 species of parasites; and the species coverage spanned major taxonomic groups, including 74 species of protozoan parasites and 206 species of worms (species information is available in Supplementary Table S1). Genomes were accompanied by functional annotations where available, including coding sequences and metadata such as genome size and assembly statistics. This extensive coverage rendered the database suitable for focused research on specific parasite groups.

### The overall workflow of PGIP

**Fig. 1:**
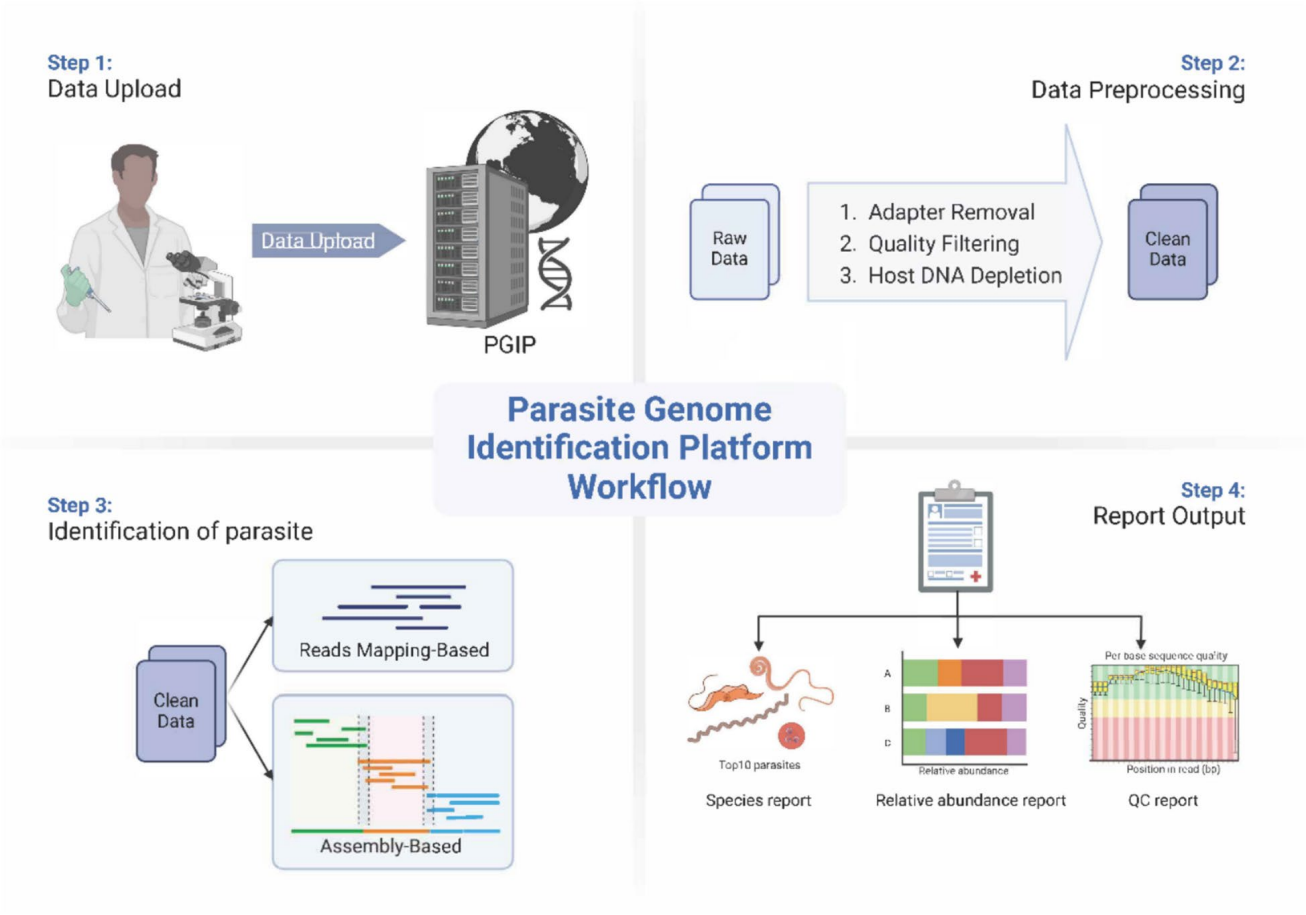
The workflow of Parasite Genome Identification Platform (PGIP). Users can upload sequencing files to PGIP, which automatically initiates the analysis and generates a report detailing the ten most abundant parasites

To initiate a workflow, users upload sequencing data to PGIP, which automatically initiates data preprocessing. This process includes adapter removal, filtering of low-quality reads, and the removal of host DNA sequences. The cleaned data then undergoes parasite species identification and analysis based on read mapping and assembly. Finally, PGIP integrates the analysis results and ranks the ten most abundant parasite species; providing species names, relative abundance, and quality control reports. A final report is generated and delivered through an automated web interface (Fig. 1).

### Web server implementation

**Fig. 2:**
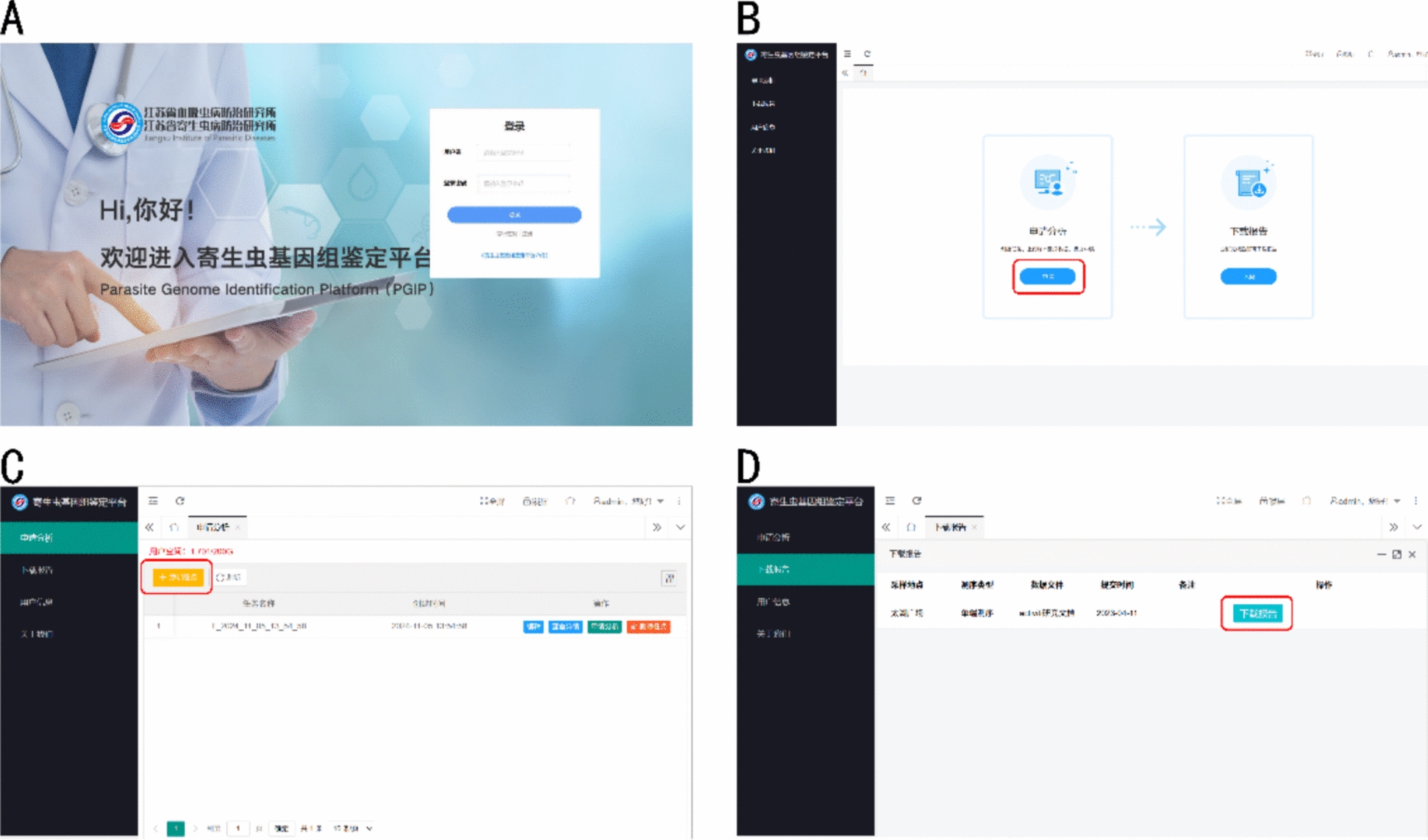
The PGIP web interface: **A** The platform login interface of PGIP. Users register to manage their personal data and analysis reports. **B** Main graphical user interface of the PGIP; which is mainly composed of two sections: analysis and report download. **C** Parasite genome identification module interface. Users can upload parasite genome sequencing data for identification by clicking the upload button. **D** Report download. Once the analysis is complete, users can download a report by clicking the download button. Additional details can be found in File. S3

We implemented a cloud-based analytical framework utilizing Java Spring Boot architecture (publicly accessible at: https://pgip.jipd.com:1443/f/login), which is designed for streamlined parasite genome identification (Fig. 2).

The operational workflow is composed of three streamlined steps: (1) upload of raw sequencing data in a standardized FASTQ format, (2) metadata annotation through structured sample information fields, and (3) single-click initiation of analytical workflows via the submission interface and obtaining identification results. The platform incorporates an intuitive browser-based console with context-sensitive guidance modules, specifically engineered to lower technical barriers for users lacking training in bioinformatics.

The PGIP backend employed a distributed architecture, with a main server responsible for the web server, file system, and database; while multiple worker servers are tasked with running jobs. The servers were set up on the Hyper Converged Infrastructure platform at the Jiangsu Institute of Parasitic Diseases.

### Evaluation of parasite identification

For all test sequencing data, including both public and in-house sequencing data, PGIP successfully identified the expected parasite species using the read-mapping-based identification module (Table 1) and reported the most abundant parasite species along with their relative abundance. Although parasitic sequences were detected in the negative control (sample 15), their relative abundance remained minimal (all < 1%), likely due to interference from homologous sequences. Consequently, these samples were deemed negative for parasitic infection.

**Table 1:** Performance evaluation of the Parasite Genome Identification Platform (PGIP)

ID	Parasite species	Data type	Sample type	Total reads	Mapped reads	Relative abundance (%)
1	*Plasmodium vivax*	Whole genome sequencing	Public dataset	51,353,148	424,506	81.26
2	*Entamoeba histolytica*	Whole genome sequencing	Public dataset	1520	7	70.00
3	*Angiostrongylus cantonensis*	Whole genome sequencing	Public dataset	73,690,202	26,143,152	70.96
4	*Clonorchis sinensis*	Whole genome sequencing	Public dataset	177,024,540	84,209,848	95.15
5	*Ascaris lumbricoides*	Whole genome sequencing	Public dataset	107,342,836	14,093,901	77.50
6	*Schistosoma japonicum*	Whole genome sequencing	Public dataset	8,929,720	4,035,842	90.69
7	*Schistosoma haematobium*	Whole genome sequencing	Public dataset	24,803,138	5,604,571	76.82
8	*Enterobius vermicularis*	Whole genome sequencing	Public dataset	1,048,780	408,194	92.65
9	*Toxoplasma gondii*	Whole genome sequencing	Public dataset	92,256,308	2,373,654	59.23
10	*Clonorchis sinensis*	Metagenomic sequencing	Stool	46,487,264	22,037	46.00
11	*Plasmodium falciparum*	Metagenomic sequencing	Whole blood	82,875,966	1,169,115	69.79
12	*Naegleria fowleri*	Metagenomic sequencing	Cerebrospinal fluid	2,586,082	50,410	29.40
13	*Spirometra erinaceieuropaei*	Metagenomic sequencing	Parasitic sample	61,488,424	28,584,220	93.37
14	*Plasmodium falciparum*	Amplicon sequencing	Amplicon	3,492,502	2,632,974	78.32
15	*Spirometra erinaceieuropaei*	Metagenomic sequencing	Whole blood	67,928,034	27,119	0.85

The report can provide valuable diagnostic clues for low-density or rare infections, as well as clinically challenging parasitic diseases (the platform-generated report is shown in Supplementary File S4).

### Test case: a sparganum infection with a difficult morphological identification

A 46-year-old male patient was treated at the Affiliated Outpatient Department of the Jiangsu Institute of Parasitic Diseases and presented with a leg mass and a predicted parasite infection. Sparganosis was suspected on the basis of his medical history, blood profile, and clinical symptoms. Although visible parasites were surgically removed, the tissue could not be morphologically identified as sparganum. Consequently, mNGS was performed on the parasitized tissue.

The extracted parasite was processed using the QIAGEN QIAamp DNA Mini Kit for total nucleic acid extraction. Following quality control, the sample was randomly fragmented with a Covaris ultrasonic disruptor. Library preparation involved end repair, A-tailing, adapter ligation, fragment selection, PCR amplification, and purification. Sequencing was performed using an Illumina HiSeq X10 platform, and yielded an average of 10 gigabases of raw data. Nucleic acid extraction and quality control was conducted by the Jiangsu Institute of Parasitic Diseases, while library construction and sequencing were completed by Beijing Novogene Bioinformatics Technology Co., Ltd.

The sequencing data were uploaded to PGIP for metagenomic analysis. The report indicated a total of 61,488,424 reads, of which 28,584,220 reads were identified as *Spirometra erinaceieuropaei* sequences, with a relative abundance of 93.37%; thus, confirming sparganum infection. The three most prevalent parasite species detected were *Spirometra erinaceieuropaei* (93.37%), *Schistocephalus solidus* (0.21%), and *Diphyllobothrium latum* (0.08%); all belonging to the subclass Cestoda, under the order Pseudophyllidea (Table 2). The PGIP analysis confirmed the diagnosis of sparganum infection, thus providing critical diagnostic information for subsequent treatment (Supplementary File S5).

**Table 2:** Ten most abundant parasites of sequencing data identified by PGIP

Rank	Parasite species	Mapped reads	Relative abundance (%)
1	*Spirometra erinaceieuropaei*	28,584,220	93.37
2	*Schistocephalus solidus*	6,5354	0.21
3	*Dibothriocephalus latus*	2,3176	0.08
4	*Trichobilharzia regenti*	2965	0.01
5	*Fasciola hepatica*	1864	0.01
6	*Schistosoma japonicum*	1614	0.01
7	*Schistosoma margrebowiei*	2894	0.01
8	*Macrostomum lignano*	1543	0.01
9	*Schmidtea mediterranea*	1583	0.01
10	*Echinostoma caproni*	1788	0.01

## Discussion

Our study introduces an online genomic analysis platform that is tailored for professionals lacking a bioinformatics background; and is designed to simplify parasite identification in metagenomic data. The platform incorporates a curated parasite genome reference database encompassing 280 clinically relevant parasite species. PGIP integrates an optimized bioinformatics pipeline that enables efficient analysis of metagenomic sequencing data while maintaining compatibility with amplicon sequencing data for precise targeted species identification. Featuring an intuitive graphical user interface, the platform implements a streamlined one-click workflow from raw data upload to automated report generation; thus significantly enhancing diagnostic efficiency and operational accessibility.

Reference databases are critical for metagenomic sequencing-based species identification; however, existing resources frequently include redundant or low-quality sequences, thereby constraining their utility for precise taxonomic classification. Broad-spectrum pathogen databases (e.g., NCBI RefSeq, GenBank) frequently contain redundant or insufficiently curated sequences, resulting in misclassifications, especially for parasites with high genomic diversity [32]. At present, there is no specialized reference genome database for parasites in the field of parasite identification. PGIP addressed this by curating a high-quality database of 280 parasite species, which are rigorously filtered for assembly completeness, annotation accuracy, and taxonomic validity. The database integrates highly specialized reference genomes for parasites, developed by researchers with expertise in parasitic disease prevention and control. This specialization enables the precise identification of unknown parasite genomes, which are supported by detailed taxonomic reports and genomic interpretations. Furthermore, PGIP’s quarterly updates ensure timely integration of newly sequenced species, thereby addressing the rapidly evolving landscape of parasite genomics [5].

Current computational platforms often rely on a single-algorithm methodology, limiting their adaptability to diverse genomic contexts. For instance, Kraken2 [26] achieves rapid classification but exhibits limited accuracy for novel species, while MetaBAT2 [29] resolves complex communities but requires high sequencing depth. Simultaneously, the assembly pipeline MEGAHIT + MetaBAT2 reconstructs genomes for low-abundance or divergent parasites, thus addressing limitations of reads mapping [10]. This hybrid methodology aligns with proposals advocating the implementation of combined strategies to enhance sensitivity and specificity in pathogen detection [7].

Most existing tools, such as Kraken2 and MetaPhlAn3 [33, 34], require command-line operations and manual preprocessing (e.g., adapter trimming, host DNA depletion, database construction); thus posing substantial barriers for clinicians and nonbioinformaticians. For instance, while Kraken2 excels in taxonomic classification speed, its dependency on Linux environments and complex parameter tuning limits its adoption in time-sensitive clinical settings [34]. In contrast, PGIP integrates an end-to-end workflow within an intuitive web interface, automating data preprocessing, analysis, and report generation. This prioritization of user-centric design directly addresses workflow simplification imperatives within diagnostic metagenomics [15, 35].

As a cloud-based online service platform, the computational capacity and storage infrastructure of PGIP are foundational components that directly influence service scalability, data throughput efficiency, and real-time analytical reliability. PGIP was deployed on the hyper-converged infrastructure of the Jiangsu Institute of Parasitic Diseases, enabling scalable high-performance computing capabilities and a stable operational environment. For example, 20 Gb of sequencing data can be processed and identified within 1.5 h.

The platform exhibited stable performance across samples with varying levels of host background interference. For example, PGIP successfully detected *Clonorchis sinensis* from stool samples (high background), *Plasmodium falciparum* from blood metagenomic data (moderate background), and *Naegleria fowleri* from cerebrospinal fluid (low background), demonstrating robust sensitivity under different sequencing complexities. The platform also accurately identified *Spirometra erinaceieuropaei* from a tissue sample with limited host background, further supporting its adaptability to diverse sample types and quality.

Beyond high-prevalence parasites such as *Plasmodium* and *Schistosoma*, PGIP proved valuable in identifying rare or diagnostically challenging infections. In a suspected case of amebic meningitis, PGIP detected *Naegleria fowleri* with a relative abundance of 29.4% in cerebrospinal fluid. This case illustrates the clinical utility of PGIP as a complementary diagnostic tool, particularly when conventional microscopy or targeted PCR yield inconclusive results.

While PGIP demonstrates notable advantages, limitations persist. Firstly, its performance relies heavily on existing parasite genome databases. In recognition of this, for the optimization of future platforms, regular updates to the database and expansion of species coverage will incorporate genome information from newly sequenced novel parasites. Secondly, the unbiased nature of metagenomic analysis introduces challenges in processing high-noise or complex mixed samples; which can potentially lead to missed detection and false positives. Currently, no universally accepted criteria exist to define a positive parasitic diagnosis based solely on mNGS read counts or relative abundance. In practice, parasite identification typically needs to be integrated with clinical presentation, epidemiological exposure, host immune status, and orthogonal testing (e.g., microscopy, serology, PCR). PGIP focuses on species-specific identification of parasitic infections, and we recognize the importance of evaluating performance on metagenomic datasets.

Future research will focus on expanding database coverage, optimizing algorithmic frameworks, and enhancing the platform’s ability to identify complex samples—particularly those with low parasite abundance or high background noise. To this end, we have initiated collaborations with established pan-pathogen metagenomic platforms, to jointly validate the platform’s utility in broader clinical contexts. These efforts aim to further elevate PGIP’s diagnostic robustness and practical value for public health and clinical applications.

## Conclusions

PGIP addressed the critical need for accessible parasite genome identification by integrating a specialized reference database with automated bioinformatics workflows. The platform demonstrated high accuracy in detecting both high-prevalence and rare parasites. Its user-friendly design lowers the technical barrier for clinicians and public health workers, thus enabling rapid integration of mNGS into routine diagnostics. With continuous advancements in algorithmic sophistication and genomic data resources, PGIP is positioned to evolve into a pivotal tool for global surveillance and control of parasitic diseases.

## Supplementary Information

Additional file 1: Table. S1. List of parasites species.

Additional file 2: File.S2. Datasets for evaluation of parasite identification.

Additional file 3: File.S3. Parasite Genome Identification Platform User Manual.

Additional file 4: File.S4. The platform report of test sequencing data.

Additional file 5: File.S5. PGIP report template.

## Data Availability

Following approval by the Jiangsu Institute of Parasitic Diseases (Wuxi, China), the datasets underlying the results of this article will be made available to investigators. PGIP publicly accessible at: https://pgip.jipd.com:1443/f/login.Please email the corresponding author for more information.

## Abbreviations

ENA: European Nucleotide Archive
MAGs: Metagenome-assembled genomes
mNGS: Metagenomic next-generation sequencing
NCBI: National Center for Biotechnology Information
ORFs: Aligning open reading frames
PGIP: Parasite Genome Identification Platform
QC: Quality control
RBAC: Role-based access control
SdBG: Simplified de Bruijn graphs
TNF: Tetranucleotide frequency
