# Supplementary material for: PGIP: a web server for the rapid taxonomic identification of parasite genomes
Source: Parasit Vectors. 2025 Aug 28;18:365. doi: 10.1186/s13071-025-07007-3 (PMC12392538; doi:10.1186/s13071-025-07007-3)
Supplement: Supplementary file 1 — Additional file 1: Table. S1. List of parasites species. [file 13071_2025_7007_MOESM1_ESM.pdf]

List of parasites species

Protozoan					
ID	Species Name	Taxonomic rank	Genome size	Total ungapped length	Assembly level
1	*Acanthamoeba castellanii*	Species	42 Mb	39.4 Mb	Scaffold
2	*Babesia bigemina*	Species	13.8 Mb	13.8 Mb	Chromosome
3	*Babesia bovis*	Species	8.2 Mb	8.2 Mb	Chromosome
4	*Babesia microti*	Species	6.4 Mb	6.4 Mb	Chromosome
5	*Babesia ovata*	Species	14.5 Mb	14.5 Mb	Contig
6	*Babesia_sp. Xinjiang*	Species	8.4 Mb	8.4 Mb	Scaffold
7	*Besnoitia besnoiti*	Species	58.8 Mb	58.8 Mb	Chromosome
8	*Blastocistis hominis*	Species	18.8 Mb	18.7 Mb	Scaffold
9	*Blastocystis hominis*	Species	18.8 Mb	18.7 Mb	Scaffold
10	*Blastocystis_sp._subtype 4*	Species	12.9 Mb	12.9 Mb	Scaffold
11	*Cryptosporidium hominis*	Species	8.7 Mb	8.7 Mb	Scaffold
12	*Cryptosporidium muris*	Species	9.2 Mb	9.2 Mb	Scaffold
13	*Cryptosporidium parvum*	Species	9.1 Mb	9.1 Mb	Chromosome
14	*Cryptosporidium ubiquitum*	Species	9 Mb	9 Mb	Scaffold
15	*Cyclospora cayetanensis*	Species	44.4 Mb	44.4 Mb	Contig
16	*Dictyostelium discoideum*	Species	34.1 Mb	34.1 Mb	Chromosome
17	*Dictyostelium purpureum*	Species	33 Mb	32.9 Mb	Scaffold
18	*Eimeria acervulina*	Species	45.8 Mb	45.7 Mb	Scaffold
19	*Eimeria maxima*	Species	46 Mb	45.9 Mb	Scaffold
20	*Eimeria mitis*	Species	72.2 Mb	66.9 Mb	Scaffold
21	*Eimeria necatrix*	Species	55 Mb	54.9 Mb	Scaffold
22	*Eimeria tenella*	Species	51.9 Mb	51.2 Mb	Scaffold
23	*Entamoeba dispar*	Species	30.6 Mb	30.5 Mb	Scaffold
24	*Entamoeba histolytica*	Species	20.8 Mb	20.8 Mb	Scaffold
25	*Entamoeba invadens*	Species	40.9 Mb	40.5 Mb	Scaffold
26	*Entamoeba nuttalli*	Species	14.4 Mb	14.4 Mb	Scaffold
27	*Giardia duodenalis*	Species	12.1 Mb	11.7 Mb	Chromosome
28	*Giardia intestinalis*	Species	12.1 Mb	11.7 Mb	Chromosome
29	*Gregarina niphandrodes*	Species	14 Mb	13.6 Mb	Scaffold
30	*Hammondia hammondi*	Species	51.5 Mb	51.5 Mb	Contig
31	*Ichthyophthirius multifiliis*	Species	48.8 Mb	48.7 Mb	Scaffold
32	*Leishmania braziliensis*	Species	32.1 Mb	32 Mb	Chromosome
33	*Leishmania donovani*	Species	32.4 Mb	31.3 Mb	Chromosome
34	*Leishmania infantum*	Species	32.1 Mb	32.1 Mb	Chromosome
35	*Leishmania major*	Species	32.9 Mb	32.9 Mb	Complete Genome

36	*Leishmania mexicana*	Species	32.1 Mb	32.1 Mb	Chromosome
37	*Leishmania panamensis*	Species	33.9 Mb	33.9 Mb	Complete Genome
38	*Leptomonas pyrrhocoris*	Species	30.4 Mb	30.3 Mb	Scaffold
39	*Naegleria fowleri*	Species	29.5 Mb	29.5 Mb	Scaffold
40	*Naegleria gruberi*	Species	41 Mb	36.3 Mb	Scaffold
41	*Neospora caninum*	Species	57.5 Mb	57.5 Mb	Chromosome
42	*Paramecium tetraurelia*	Species	72.1 Mb	71.5 Mb	Scaffold
43	*Perkinsus marinus*	Species	86.6 Mb	86 Mb	Scaffold
44	*Plasmodium berghei*	Species	18.7 Mb	18.7 Mb	Chromosome
45	*Plasmodium chabaudi*	Species	18.9 Mb	18.9 Mb	Chromosome
46	*Plasmodium coatneyi*	Species	27.7 Mb	27.7 Mb	Chromosome
47	*Plasmodium cynomolgi*	Species	26.2 Mb	25.3 Mb	Chromosome
48	*Plasmodium falciparum*	Species	23.3 Mb	23.3 Mb	Complete Genome
49	*Plasmodium fragile*	Species	25.9 Mb	22.9 Mb	Scaffold
50	*Plasmodium gaboni*	Species	20.4 Mb	19.9 Mb	Chromosome
51	*Plasmodium gallinaceum*	Species	25 Mb	23.8 Mb	Scaffold
52	*Plasmodium gonderi*	Species	33 Mb	32.9 Mb	Scaffold
53	*Plasmodium inui*	Species	27.4 Mb	26.2 Mb	Scaffold
54	*Plasmodium knowlesi*	Species	24.4 Mb	24.3 Mb	Chromosome
55	*Plasmodium malariae*	Species	33.6 Mb	33.6 Mb	Chromosome
56	*Plasmodium reichenowi*	Species	20.5 Mb	19.7 Mb	Chromosome
57	*Plasmodium relictum*	Species	22.6 Mb	22.5 Mb	Chromosome
58	*Plasmodium vinckei*	Species	18.3 Mb	18.3 Mb	Chromosome
59	*Plasmodium vivax*	Species	27 Mb	27 Mb	Chromosome
60	*Plasmodium yoelii*	Species	23 Mb	23 Mb	Complete Genome
61	*Plasmodium_sp._gorilla_clade G2*	Species	22.2 Mb	22.2 Mb	Chromosome
62	*Tetrahymena thermophila*	Species	103 Mb	103 Mb	Scaffold
63	*Theileria annulata*	Species	8.4 Mb	8.4 Mb	Chromosome
64	*Theileria equi*	Species	11.6 Mb	11.6 Mb	Chromosome
65	*Theileria orientalis*	Species	9 Mb	9 Mb	Chromosome
66	*Theileria parva*	Species	8.3 Mb	8.3 Mb	Chromosome
67	*Toxoplasma gondii*	Species	65.6 Mb	65.4 Mb	Chromosome
68	*Trichomonas vaginalis*	Species	181.5 Mb	181.4 Mb	Chromosome
69	*Trypanosoma brucei*	Species	22.1 Mb	22.1 Mb	Chromosome
70	*Trypanosoma conorhini*	Species	21.3 Mb	21.1 Mb	Scaffold
71	*Trypanosoma cruzi*	Species	89.9 Mb	89.6 Mb	Scaffold
72	*Trypanosoma grayi*	Species	20.9 Mb	20.8 Mb	Scaffold
73	*Trypanosoma rangeli*	Species	21.2 Mb	21 Mb	Scaffold
74	*Trypanosoma theileri*	Species	29.8 Mb	25.7 Mb	Scaffold

**Worms**					
ID	Species Name	Taxonomic rank	Genome size	Total ungapped length	Assembly level
1	*Acanthocheilonema viteae*	Species	81.1 Mb	81.1 Mb	Chromosome
2	*Acrobeloides nanus*	Species	188.9 Mb	188.9 Mb	Chromosome
3	*Allodiplogaster sudhausi*	Species	352.2 Mb	352.1 Mb	Scaffold
4	*Ancylostoma caninum*	Species	348 Mb	348 Mb	Scaffold
5	*Ancylostoma ceylanicum*	Species	313.1 Mb	300.9 Mb	Scaffold
6	*Ancylostoma duodenale*	Species	319.2 Mb	319.1 Mb	Chromosome
7	*Angiostrongylus cantonensis*	Species	293.3 Mb	273.6 Mb	Scaffold
8	*Angiostrongylus costaricensis*	Species	262.8 Mb	262.1 Mb	Scaffold
9	*Angiostrongylus vasorum*	Species	279.9 Mb	279.9 Mb	Scaffold
10	*Anisakis simplex*	Species	126.9 Mb	122.9 Mb	Scaffold
11	*Aphelenchoides besseyi*	Species	47.4 Mb	47.4 Mb	Scaffold
12	*Aphelenchoides bicaudatus*	Species	46.4 Mb	46.4 Mb	Contig
13	*Aphelenchoides fujianensis*	Species	143.8 Mb	143.8 Mb	Scaffold
14	*Ascaris lumbricoides*	Species	296 Mb	295 Mb	Scaffold
15	*Ascaris suum*	Species	278.6 Mb	278.6 Mb	Chromosome
16	*Atriophallophorus winterbourni*	Species	601.7 Mb	599.4 Mb	Scaffold
17	*Auanema sp. JU1783*	Species	59.7 Mb	53.9 Mb	Scaffold
18	*Bradynema listronoti*	Species	80.5 Mb	80.5 Mb	Scaffold
19	*Brugia malayi*	Species	87.2 Mb	86.9 Mb	Scaffold
20	*Brugia pahangi*	Species	96.4 Mb	96.4 Mb	Chromosome
21	*Brugia timori*	Species	64.9 Mb	64.2 Mb	Scaffold
22	*Bunonema rgd898*	Species	34.1 Mb	34 Mb	Scaffold
23	*Bursaphelenchus okinawaensis*	Species	70 Mb	70 Mb	Scaffold
24	*Bursaphelenchus xylophilus*	Species	78.3 Mb	78.3 Mb	Scaffold
25	*Caenorhabditis angaria*	Species	72 Mb	72 Mb	Chromosome
26	*Caenorhabditis becei*	Species	93.9 Mb	93.9 Mb	Chromosome
27	*Caenorhabditis bovis*	Species	62.7 Mb	62.7 Mb	Contig
28	*Caenorhabditis brenneri*	Species	126.5 Mb	126.5 Mb	Chromosome
29	*Caenorhabditis briggsae*	Species	108.4 Mb	105.4 Mb	Chromosome
30	*Caenorhabditis elegans*	Species	100.3 Mb	100.3 Mb	Complete Genome
31	*Caenorhabditis inopinata*	Species	123 Mb	122.6 Mb	Chromosome
32	*Caenorhabditis japonica*	Species	152 Mb	152 Mb	Chromosome
33	*Caenorhabditis latens*	Species	120.4 Mb	120.4 Mb	Chromosome
34	*Caenorhabditis nigoni*	Species	128.5 Mb	128.5 Mb	Chromosome
35	*Caenorhabditis panamensis*	Species	79 Mb	78.1 Mb	Scaffold
36	*Caenorhabditis parvicauda*	Species	115.3 Mb	115.2 Mb	Chromosome
37	*Caenorhabditis quiockensis*	Species	114.8 Mb	114.8 Mb	Chromosome
38	*Caenorhabditis remanei*	Species	130.5 Mb	130.5 Mb	Chromosome

39	*Caenorhabditis sinica*	Species	152.4 Mb	152.4 Mb	Chromosome
40	*Caenorhabditis sulstoni*	Species	76.3 Mb	76.3 Mb	Chromosome
41	*Caenorhabditis tribulationis*	Species	101.2 Mb	101.1 Mb	Scaffold
42	*Caenorhabditis tropicalis*	Species	82.2 Mb	82.2 Mb	Chromosome
43	*Caenorhabditis uteleia*	Species	114.3 Mb	114.3 Mb	Chromosome
44	*Caenorhabditis waitukubuli*	Species	76.1 Mb	76.1 Mb	Chromosome
45	*Caenorhabditis zanzibari*	Species	107.2 Mb	107.2 Mb	Chromosome
46	*Cercopithifilaria johnstoni*	Species	76.9 Mb	76.9 Mb	Scaffold
47	*Clonorchis sinensis*	Species	558.1 Mb	555.2 Mb	Chromosome
48	*Cylicocyclus nassatus*	Species	514.7 Mb	513.9 Mb	Scaffold
49	*Cylicostephanus goldi*	Species	173.4 Mb	164.8 Mb	Scaffold
50	*Dibothriocephalus latus*	Species	531.4 Mb	493.1 Mb	Scaffold
51	*Dicrocoelium dendriticum*	Species	1.9 Gb	1.9 Gb	Chromosome
52	*Dictyocaulus viviparus*	Species	181.1 Mb	181 Mb	Chromosome
53	*Diplogasteroides magnus*	Species	214.2 Mb	213.8 Mb	Scaffold
54	*Diploscapter coronatus*	Species	169.7 Mb	169.7 Mb	Chromosome
55	*Diploscapter pachys*	Species	157.7 Mb	156.5 Mb	Scaffold
56	*Dirofilaria immitis*	Species	86.8 Mb	86.8 Mb	Contig
57	*Ditylenchus destructor*	Species	133.8 Mb	133.8 Mb	Chromosome
58	*Ditylenchus dipsaci*	Species	227.2 Mb	227.2 Mb	Scaffold
59	*Dracunculus medinensis*	Species	103.8 Mb	103.6 Mb	Scaffold
60	*Echinococcus canadensis*	Species	115 Mb	115 Mb	Scaffold
61	*Echinococcus granulosus*	Species	110.8 Mb	110.1 Mb	Scaffold
62	*Echinococcus multilocularis*	Species	115 Mb	112.1 Mb	Scaffold
63	*Echinococcus oligarthrus*	Species	106 Mb	106 Mb	Contig
64	*Echinostoma caproni*	Species	834.6 Mb	769.2 Mb	Scaffold
65	*Elaeophora elaphi*	Species	1.5 Mb	1.5 Mb	Scaffold
66	*Enoplolaimus lenunculus*	Species	300.9 Mb	300.9 Mb	Scaffold
67	*Enterobius vermicularis*	Species	150.1 Mb	144.2 Mb	Scaffold
68	*Epsilonema sp. ZAB3_2*	Species	171.9 Mb	171.9 Mb	Contig
69	*Fasciola gigantica*	Species	879.1 Mb	850.8 Mb	Scaffold
70	*Fasciola hepatica*	Species	1.5 Gb	1.5 Gb	Contig
71	*Fasciolopsis buski*	Species	748.2 Mb	720.1 Mb	Scaffold
72	*Globodera pallida*	Species	113.2 Mb	112.3 Mb	Scaffold
73	*Globodera rostochiensis*	Species	92.7 Mb	92.2 Mb	Scaffold
74	*Gongylonema pulchrum*	Species	322.3 Mb	302.4 Mb	Scaffold
75	*Gyrodactylus bullatarudis*	Species	84.3 Mb	83.3 Mb	Scaffold
76	*Gyrodactylus salaris*	Species	67.4 Mb	67.3 Mb	Scaffold
77	*Haemonchus contortus*	Species	283.4 Mb	277.7 Mb	Chromosome
78	*Haemonchus placei*	Species	259.1 Mb	248.1 Mb	Scaffold
79	*Halicephalobus mephisto*	Species	61.4 Mb	61.4 Mb	Scaffold
80	*Halicephalobus sp. NKZ332*	Species	47.4 Mb	47.2 Mb	Scaffold
81	*Heligmosomoides polygyrus*	Species	649.1 Mb	648.5 Mb	Chromosome

82	*Heterobilharzia americana*	Species	665.6 Mb	665.2 Mb	Chromosome
83	*Heterodera glycines*	Species	158 Mb	156.3 Mb	Chromosome
84	*Heterodera schachtii*	Species	179.2 Mb	174.3 Mb	Scaffold
85	*Heterorhabditis bacteriophora*	Species	86.3 Mb	86.3 Mb	Chromosome
86	*Hydatigera taeniaeformis*	Species	103.7 Mb	100.5 Mb	Scaffold
87	*Hymenolepis diminuta*	Species	177.1 Mb	168 Mb	Scaffold
88	*Hymenolepis microstoma*	Species	168.9 Mb	162.4 Mb	Chromosome
89	*Hymenolepis nana*	Species	162.9 Mb	156.8 Mb	Scaffold
90	*Koerneria luziae*	Species	177.3 Mb	177.1 Mb	Scaffold
91	*Levipalatum texanum*	Species	154.9 Mb	154.4 Mb	Scaffold
92	*Linhomoeus sp. GSCO2_2*	Species	240.5 Mb	240.5 Mb	Contig
93	*Litomosoides sigmodontis*	Species	65.9 Mb	65.9 Mb	Chromosome
94	*Loa loa*	Species	91.4 Mb	87.5 Mb	Scaffold
95	*Macrostomum lignano*	Species	764.4 Mb	762.8 Mb	Scaffold
96	*Meloidogyne arenaria*	Species	315 Mb	314.9 Mb	Scaffold
97	*Meloidogyne chitwoodi*	Species	47.5 Mb	47.5 Mb	Contig
98	*Meloidogyne enterolobii*	Species	285.4 Mb	285.4 Mb	Contig
99	*Meloidogyne floridensis*	Species	74.8 Mb	74.6 Mb	Scaffold
100	*Meloidogyne graminicola*	Species	39.2 Mb	39.2 Mb	Scaffold
101	*Meloidogyne hapla*	Species	59.2 Mb	59.1 Mb	Chromosome
102	*Meloidogyne incognita*	Species	225 Mb	225 Mb	Scaffold
103	*Meloidogyne javanica*	Species	301.8 Mb	301.7 Mb	Scaffold
104	*Mesocestoides corti*	Species	117.2 Mb	116.3 Mb	Scaffold
105	*Mesodorylaimus sp. YZB2_4*	Species	148 Mb	148 Mb	Scaffold
106	*Mesorhabditis belari*	Species	209 Mb	208.8 Mb	Scaffold
107	*Mesorhabditis spiculigera*	Species	197.8 Mb	197.7 Mb	Scaffold
108	*Micoletzkya japonica*	Species	201.9 Mb	180.4 Mb	Scaffold
109	*Microlaimidae sp. YZB2_3*	Species	575.8 Mb	575.8 Mb	Contig
110	*Necator americanus*	Species	234.5 Mb	234.2 Mb	Chromosome
111	*Nippostrongylus brasiliensis*	Species	257.4 Mb	257.2 Mb	Chromosome
112	*Oesophagostomum dentatum*	Species	443 Mb	351.6 Mb	Scaffold
113	*Onchocerca flexuosa*	Species	67.7 Mb	67.7 Mb	Scaffold
114	*Onchocerca ochengi*	Species	91.7 Mb	91.3 Mb	Scaffold
115	*Onchocerca volvulus*	Species	96.3 Mb	93.3 Mb	Scaffold
116	*Opisthorchis felineus*	Species	679.2 Mb	607 Mb	Scaffold
117	*Opisthorchis viverrini*	Species	620.5 Mb	572.1 Mb	Scaffold
118	*Oscheius tipulae*	Species	60.4 Mb	60.4 Mb	Contig
119	*Panagrellus redivivus*	Species	65.1 Mb	62.1 Mb	Scaffold
120	*Panagrolaimus davidi*	Species	118.1 Mb	118.0 Mb	Scaffold
121	*Panagrolaimus es5*	Species	90.2 Mb	88.7 Mb	Scaffold
122	*Panagrolaimus ps1159*	Species	85 Mb	83.3 Mb	Scaffold
123	*Panagrolaimus superbus*	Species	103.1 Mb	103.1 Mb	Chromosome
124	*Paragonimus heterotremus*	Species	841.2 Mb	777.9 Mb	Scaffold

125	*Paragonimus kellicotti*	Species	696.5 Mb	616.6 Mb	Scaffold
126	*Paragonimus skrjabinimiyazakii*	Species	915.8 Mb	836.5 Mb	Scaffold
127	*Paragonimus westermani*	Species	922.8 Mb	876.4 Mb	Scaffold
128	*Paralinhomoeus sp. GSCO2_6*	Species	177.1 Mb	177.1 Mb	Scaffold
129	*Parapristionchus giblindavisi*	Species	352.1 Mb	352.1 Mb	Contig
130	*Parascaris equorum*	Species	181.4 Mb	181.4 Mb	Contig
131	*Parascaris univalens*	Species	243.7 Mb	243.6 Mb	Chromosome
132	*Parastrongyloides trichosuri*	Species	42.5 Mb	42.2Mb	Scaffold
133	*Parelaphostrongylus tenuis*	Species	490.9 Mb	490.9 Mb	Scaffold
134	*Plectus sambesii*	Species	186.7 Mb	186.6 Mb	Scaffold
135	*Pristionchus arcanus*	Species	202.8 Mb	194.5 Mb	Scaffold
136	*Pristionchus entomophagus*	Species	244.9 Mb	244.4 Mb	Scaffold
137	*Pristionchus exspectatus*	Species	169.6 Mb	169.5 Mb	Scaffold
138	*Pristionchus fissidentatus*	Species	249 Mb	248.9 Mb	Scaffold
139	*Pristionchus japonicus*	Species	223.1 Mb	198.6 Mb	Scaffold
140	*Pristionchus maxplancki*	Species	265.6 Mb	222.4 Mb	Scaffold
141	*Pristionchus mayeri*	Species	290.8 Mb	290.6 Mb	Scaffold
142	*Pristionchus pacificus*	Species	158.5 Mb	155 Mb	Chromosome
143	*Protopolystoma xenopodis*	Species	617.3 Mb	593 Mb	Scaffold
144	*Ptycholaimellus sp. GST1_10*	Species	238.3 Mb	238.3 Mb	Contig
145	*Rhabditophanes sp. KR3021*	Species	44.8 Mb	44.8 Mb	Scaffold
146	*Rhynchonema sp. JSB1_4*	Species	221.9 Mb	221.9 Mb	Contig
147	*Romanomermis culicivorax*	Species	359.1 Mb	359 Mb	Scaffold
148	*Sabatieria punctata*	Species	390.7 Mb	390.7 Mb	Scaffold
149	*Schistocephalus solidus*	Species	625.2 Mb	625.2 Mb	Scaffold
150	*Schistosoma bovis*	Species	391.8 Mb	391.7 Mb	Chromosome
151	*Schistosoma curassoni*	Species	396.9 Mb	396.8 Mb	Chromosome
152	*Schistosoma guineensis*	Species	379.3 Mb	379.2 Mb	Chromosome
153	*Schistosoma haematobium*	Species	400.3 Mb	400.2 Mb	Chromosome
154	*Schistosoma intercalatum*	Species	386.1 Mb	386 Mb	Chromosome
155	*Schistosoma japonicum*	Species	406.6 Mb	404.4 Mb	Chromosome
156	*Schistosoma mansoni*	Species	391.4 Mb	391.4 Mb	Chromosome
157	*Schistosoma margrebowiei*	Species	394.9 Mb	394.8 Mb	Chromosome
158	*Schistosoma mattheei*	Species	389.3 Mb	389.2 Mb	Chromosome
159	*Schistosoma rodhaini*	Species	388.5 Mb	388.4 Mb	Chromosome
160	*Schistosoma spindale*	Species	390.1 Mb	390 Mb	Chromosome
161	*Schistosoma turkestanicum*	Species	425 Mb	423.3 Mb	Chromosome
162	*Schmidtea mediterranea*	Species	840.2 Mb	840.2 Mb	Chromosome
163	*Setaria digitata*	Species	78.8 Mb	78.8 Mb	Scaffold
164	*Soboliphyme baturini*	Species	218.3 Mb	213 Mb	Scaffold
165	*Spirometra erinaceieuropaei*	Species	1.3 Gb	1.1 Gb	Scaffold
166	*Steinernema carpocapsae*	Species	84.5 Mb	84.1 Mb	Chromosome
167	*Steinernema feltiae*	Species	121.6 Mb	121.1 Mb	Contig

168	*Steinernema glaseri*	Species	92.8 Mb	89.5 Mb	Scaffold
169	*Steinernema hermaphroditum*	Species	90.7 Mb	90.7 Mb	Chromosome
170	*Steinernema monticolum*	Species	89.2 Mb	84.8 Mb	Scaffold
171	*Steinernema scapterisci*	Species	79.7 Mb	78.9 Mb	Scaffold
172	*Strongyloides papillosus*	Species	58.2 Mb	58.2 Mb	Contig
173	*Strongyloides ratti*	Species	43.2 Mb	42.9 Mb	Chromosome
174	*Strongyloides stercoralis*	Species	44.4 Mb	44.4 Mb	Chromosome
175	*Strongyloides venezuelensis*	Species	56 Mb	56 Mb	Scaffold
176	*Strongylus vulgaris*	Species	291.1 Mb	265.5 Mb	Scaffold
177	*Syphacia muris*	Species	96.2 Mb	89.3 Mb	Scaffold
178	*Taenia asiatica*	Species	168.7 Mb	164.4 Mb	Scaffold
179	*Taenia multiceps*	Species	240.6 Mb	240.5 Mb	Chromosome
180	*Taenia saginata*	Species	169.1 Mb	166.3 Mb	Scaffold
181	*Taenia solium*	Species	129.8 Mb	129.8 Mb	Scaffold
182	*Teladorsagia circumcincta*	Species	700.6 Mb	576.1 Mb	Scaffold
183	*Thelazia callipaeda*	Species	117.6 Mb	117.6 Mb	Chromosome
184	*Theristus sp. LFF4_11*	Species	169.2 Mb	169.2 Mb	Contig
185	*Toxocara canis*	Species	317.1 Mb	298.6 Mb	Scaffold
186	*Trichinella britovi*	Species	51.5 Mb	51.5 Mb	Scaffold
187	*Trichinella murrelli*	Species	64.7 Mb	64.7 Mb	Chromosome
188	*Trichinella nativa*	Species	47.2 Mb	47.1 Mb	Scaffold
189	*Trichinella nelsoni*	Species	47.4 Mb	47.2 Mb	Scaffold
190	*Trichinella papuae*	Species	46.8 Mb	46.7 Mb	Scaffold
191	*Trichinella patagoniensis*	Species	49.8 Mb	49.7 Mb	Scaffold
192	*Trichinella pseudospiralis*	Species	56.6 Mb	56.6 Mb	Contig
193	*Trichinella sp. T6*	Species	50.9 Mb	50.6 Mb	Scaffold
194	*Trichinella sp. T8*	Species	49.3 Mb	49.1 Mb	Scaffold
195	*Trichinella sp. T9*	Species	49.1 Mb	48.8 Mb	Scaffold
196	*Trichinella spiralis*	Species	63.6 Mb	58.6 Mb	Chromosome
197	*Trichinella zimbabwensis*	Species	50.8 Mb	50.5 Mb	Scaffold
198	*Trichobilharzia regenti*	Species	954.2 Mb	954.1 Mb	Chromosome
199	*Trichobilharzia szidati*	Species	1.1 Gb	1.1 Gb	Chromosome
200	*Trichuris muris*	Species	111.8 Mb	110.1 Mb	Scaffold
201	*Trichuris suis*	Species	63.8 Mb	62.9 Mb	Scaffold
202	*Trichuris trichiura*	Species	72.7 Mb	72.7 Mb	Contig
203	*Trileptium ribeirensis*	Species	746 Mb	746 Mb	Contig
204	*Trissonchulus latispiculum*	Species	792.4 Mb	792.4 Mb	Scaffold
205	*Trissonchulus sp. WLG1_4*	Species	447 Mb	447 Mb	Scaffold
206	*Wuchereria bancrofti*	Species	88.4 Mb	87 Mb	Scaffold
