# Supplementary material for: PGIP: a web server for the rapid taxonomic identification of parasite genomes
Source: Parasit Vectors. 2025 Aug 28;18:365. doi: 10.1186/s13071-025-07007-3 (PMC12392538; doi:10.1186/s13071-025-07007-3)
Supplement: Supplementary file 2 — Additional file 2: File.S2. Datasets for evaluation of parasite identification. [file 13071_2025_7007_MOESM2_ESM.pdf]

Datasets for evaluation of parasite identification

Public datasets (Sample1-9)

ID	Parasite species	SRA ID
1	*Plasmodium vivax*	SRR1759139
2	*Entamoeba histolytica*	SRR072207
3	*Angiostrongylus cantonensis*	SRR1588192
4	*Clonorchis sinensis*	SRR5807780
5	*Ascaris lumbricoides*	SRR8448496
6	*Schistosoma japonicum*	ERR310939
7	*Schistosoma haematobium*	SRR13579869
8	*Enterobius vermicularis*	ERR310935
9	*Toxoplasma gondii*	DRR513077

In-house datasets (Sample10-15)

The research samples involved in this study were signed with informed consent by patients and has obtained ethical approved from the Ethical review committee of Jiangsu Institute of Parasitic Diseases.

Stool sample (sample 10)

The sample was sourced from the Jiangsu Institute of Parasitic Diseases during the 2024 parasitic disease survey, and infection with *Clonorchis sinensis* was confirmed by microscopic examination. The stool sample was processed using the QIAGEN QIAamp Fast DNA Stool Mini Kit for total nucleic acid extraction.

Metagenomic sequencing was performed using 20 μL of extracted nucleic acid, following the steps below: the DNA was randomly fragmented with a Covaris ultrasonic disruptor. Library preparation involved end repair, A-tailing, adapter ligation, fragment selection, PCR amplification, and purification. Sequencing was performed using an Illumina HiSeq X10 platform, and yielded an average of 10 gigabases of raw data. Nucleic acid extraction and quality control was conducted by the Jiangsu Institute of Parasitic Diseases, while library construction and sequencing were completed by Beijing Novogene Bioinformatics Technology Co., Ltd.

Blood sample (sample 11)

The samples were derived from the imported malaria biobank of the Jiangsu Institute of Parasitic Diseases. The sample comprises blood from a 2023 imported *Plasmodium falciparum* case originating from Rwanda to China. *Plasmodium falciparum* infection was confirmed in the patient through microscopic examination and rapid diagnostic testing (RDT). The blood sample was processed using the QIAGEN QIAamp DNA Mini Kit for total nucleic acid extraction.

Metagenomic sequencing was performed using 20 μL of extracted nucleic acid, following the steps below: the DNA was randomly fragmented with a Covaris ultrasonic disruptor. Library preparation involved end repair, A-tailing, adapter ligation, fragment selection, PCR amplification, and purification. Sequencing was performed using an Illumina HiSeq X10 platform, and yielded an average of 10 gigabases of raw data. Nucleic acid extraction and quality control was conducted by the Jiangsu Institute of Parasitic Diseases, while library construction and sequencing were completed by Beijing Novogene Bioinformatics Technology Co., Ltd.

Cerebrospinal fluid sample (sample 12)

The cerebrospinal fluid was obtained from a patient suspected of being infected with Amoeba Negri in December 2024.The cerebrospinal fluid was processed using the QIAGEN QIAamp DNA Mini Kit for total nucleic acid extraction.

Following quality control, the sample was randomly fragmented with a Covaris ultrasonic disruptor. Library preparation involved end repair, A-tailing, adapter ligation, fragment selection, PCR amplification, and purification. Sequencing was performed using an Illumina HiSeq X10 platform, and yielded an average of 10 gigabases of raw data. Nucleic acid extraction and quality control was conducted by the Jiangsu Institute of Parasitic Diseases, while library construction and sequencing were completed by Beijing Novogene Bioinformatics Technology Co., Ltd.

Parasitic sample (sample 13)

Describe in section 'Test case: A sparganum infection with a difficult morphological identification'.

Amplicon sequencing sample (sample 14)

The samples were derived from the imported malaria biobank of the Jiangsu Institute of Parasitic Diseases. The sample comprises blood from a 2023 imported *Plasmodium falciparum* case originating from Rwanda to China. *Plasmodium falciparum* infection was confirmed in the patient through microscopic examination and rapid diagnostic testing (RDT). The blood sample was processed using the QIAGEN QIAamp DNA Mini Kit for total nucleic acid extraction.

Amplicon sequencing targeting *Plasmodium falciparum* drug-resistance gene loci (*Pfk13*, *PfCRT*, *Pfdhfr*, *PfMDR1*) was performed using 10 μL of extracted nucleic acids, following this workflow: PCR amplification with primers tailored for the BGI ATOPlex platform, purification, adapter ligation, purification, and sequencing. Sequencing was conducted on a BGI MGISEQ-200 platform, yielding an average of 200 Mb of raw sequencing data per sample. All experiments were conducted by Jiangsu Institute of parasitic diseases.

Negative sample (sample 15)

We selected a clinical blood sample known without evidence of parasitic infection (diagnosed as bacterial infection) as the negative control.

The blood sample was processed using the QIAGEN QIAamp DNA Mini Kit for total nucleic acid extraction. Metagenomic sequencing was performed using 20 μL of extracted nucleic acid, following the steps below: the DNA was randomly fragmented with a Covaris ultrasonic disruptor. Library preparation involved end repair, A-tailing, adapter ligation, fragment selection, PCR amplification, and purification. Sequencing was performed using an Illumina HiSeq X10 platform, and yielded an average of 10 gigabases of raw data. Nucleic acid extraction and quality control was conducted by the Jiangsu Institute of Parasitic Diseases, while library construction and sequencing were completed by Beijing Novogene Bioinformatics Technology Co., Ltd.
