# Supplementary material for: PGIP: a web server for the rapid taxonomic identification of parasite genomes
Source: Parasit Vectors. 2025 Aug 28;18:365. doi: 10.1186/s13071-025-07007-3 (PMC12392538; doi:10.1186/s13071-025-07007-3)
Supplement: Supplementary file 3 — Additional file 3: File.S3. Parasite Genome Identification Platform User Manual. [file 13071_2025_7007_MOESM3_ESM.pdf]

Parasitic Genome Identification Platform User Manual

Version：2507

Jiangsu Institute of Parasitic
Diseases

Contents

1 Registration 3
2 Log in 4
3 Platform homepage 4
4 Submit an application 5
5 Download the report 8
6 User Information Management 9

1 Registration

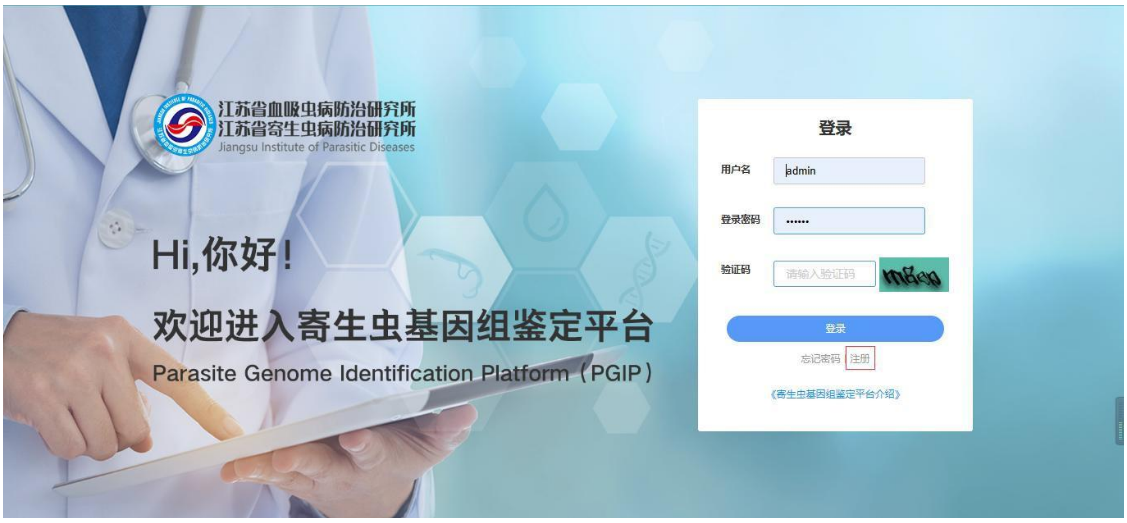

If you do not have an account on the login page, you can click on register to register as a user.

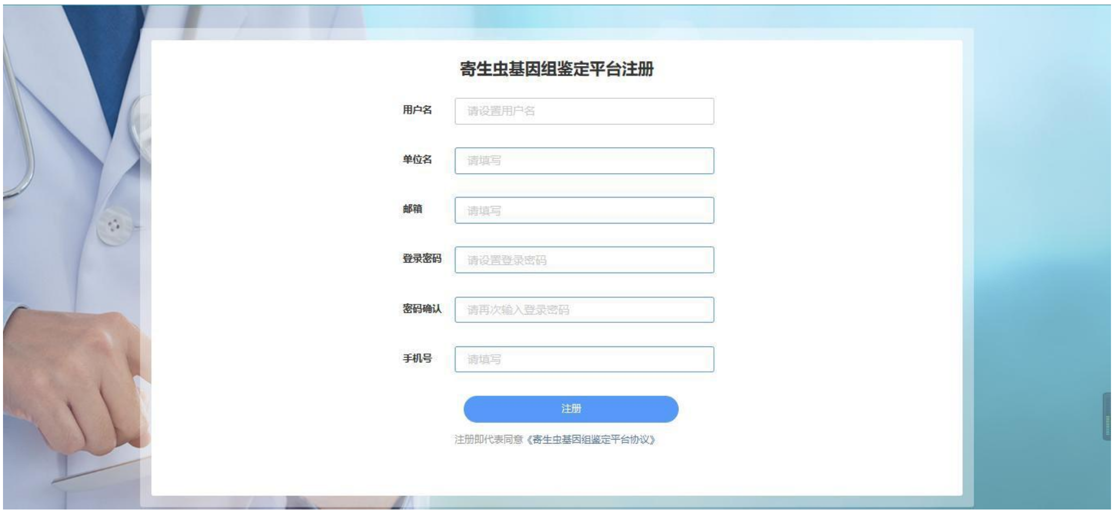

Fill in the relevant information, click register, and then you can log in using the registered account.

2 Log in

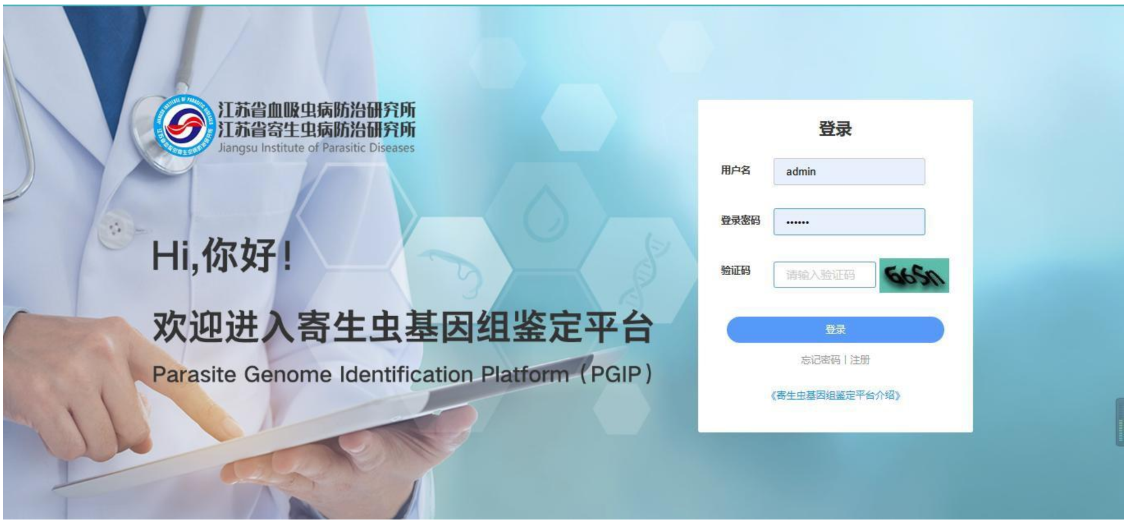

Access the login interface and enter your registered username, password, and verification code to authenticate.

3 Platform homepage

After logging in, e click the "Apply" button to enter the application analysis page, and click the "Download" button to enter the download report page.

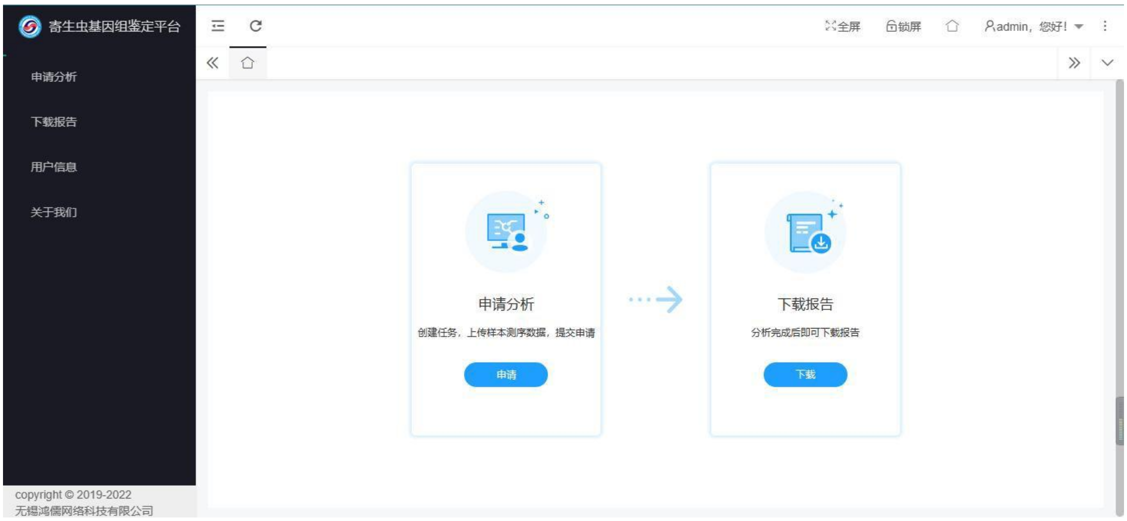

4 Submit an application

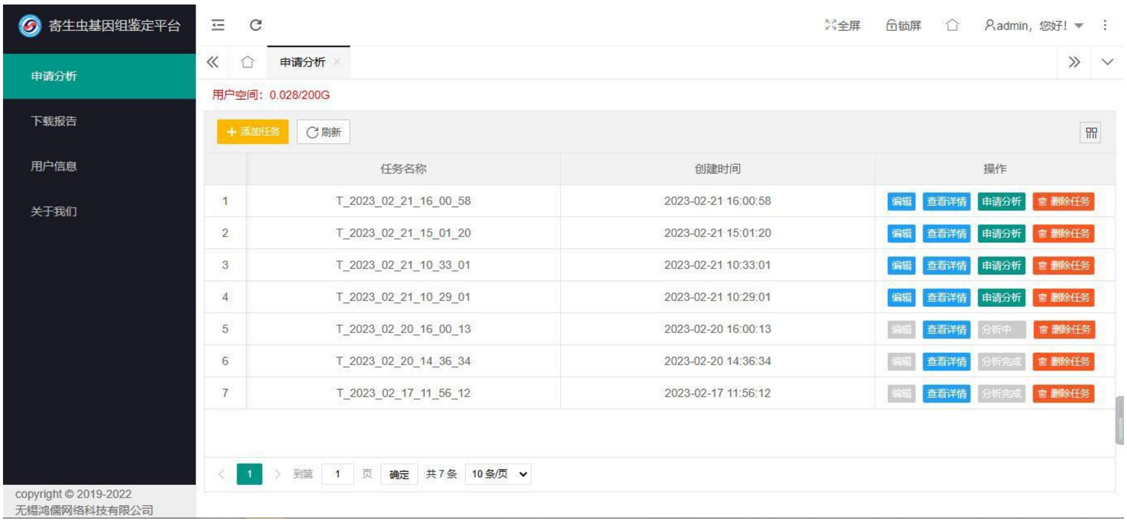

Access the application analysis interface.

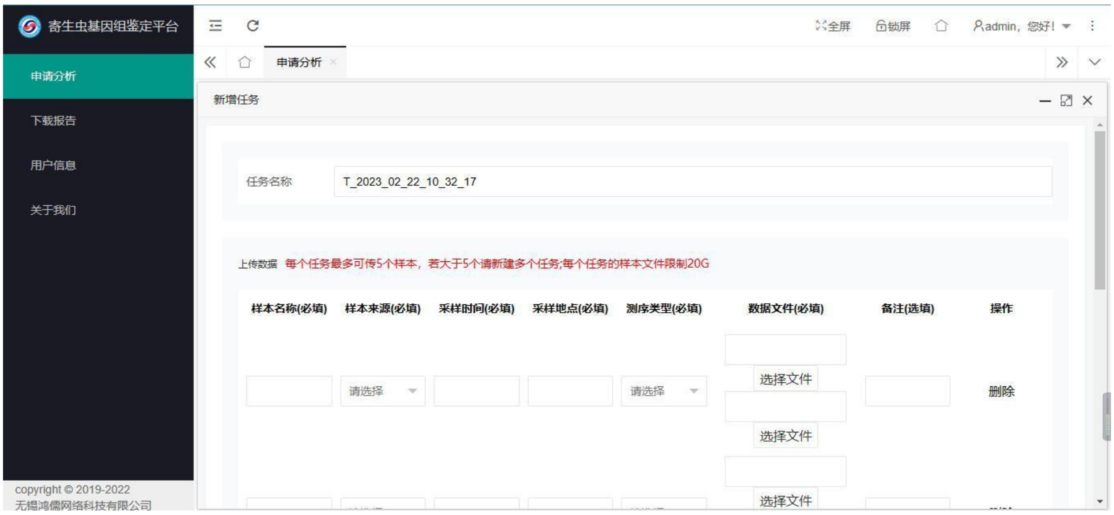

Click the "Add Task" button in the table header to access the task creation interface. The system will automatically generate a task name. Each task supports a maximum of 5 sample data uploads.

The maximum data size for each sample is 20 GB, the recommended sequencing mode is 150-bp paired-end sequencing.

Task management

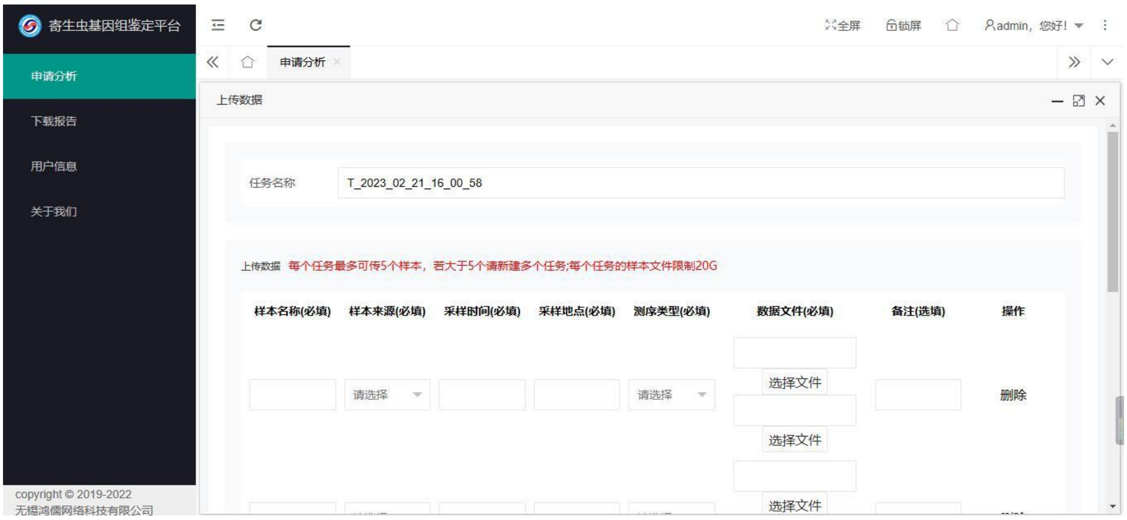

When a task has not yet been submitted for analysis, users can click the "Edit Task" button to access the task editing interface, where they may modify the sample data and associated files uploaded within the task.

Once a task has been submitted for analysis, it becomes non-editable.

View Details

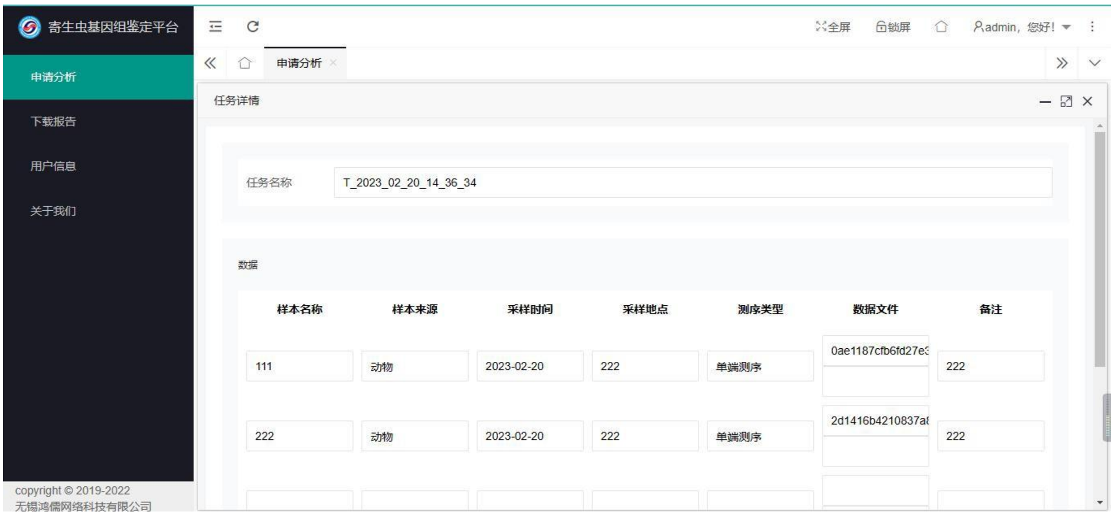

Click the "View Details" button in the list to access the details interface, where you can review the sample information associated with the task.

Task Analysis

For unanalyzed tasks, click the "Submit Analysis" button to initiate backend processing. The system will automatically analyze the task and generate an analytical report. Upon submission, the button label changes to "Analyzing" and remains disabled, reflecting the real-time task status.。

Once the backend processing is complete, the button label will update to "Analysis Completed" and is disabled to prevent re-initiation. Users may navigate to the report download interface to retrieve the analytical report generated for this task.

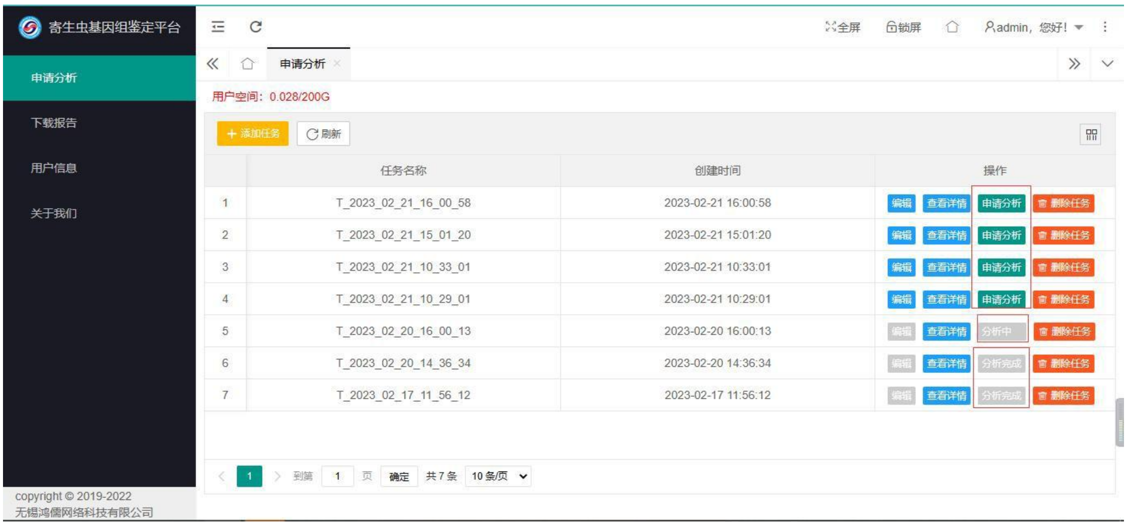

Delete Task

The "Delete Task" button enables permanent deletion of the selected task from the PGIP.

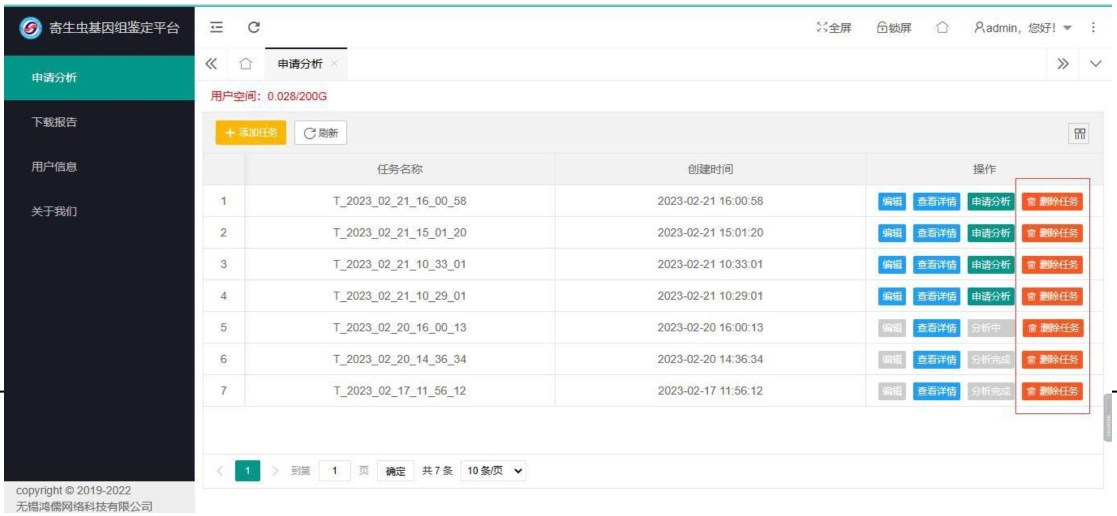

5 Download the report

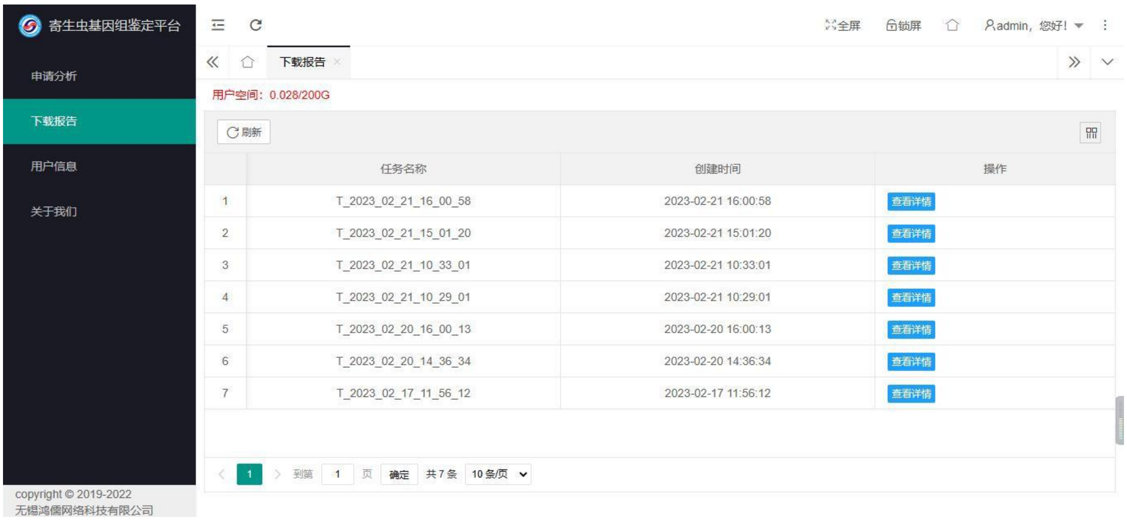

On the report download interface, users can retrieve task analysis reports generated and uploaded by the backend system.

Click the "View Details" button adjacent to each task in the list to access its dedicated details interface, where analytical reports for individual samples within the task are available for download. As illustrated in the following diagram, click the "Download Report" button to obtain the corresponding analytical report.

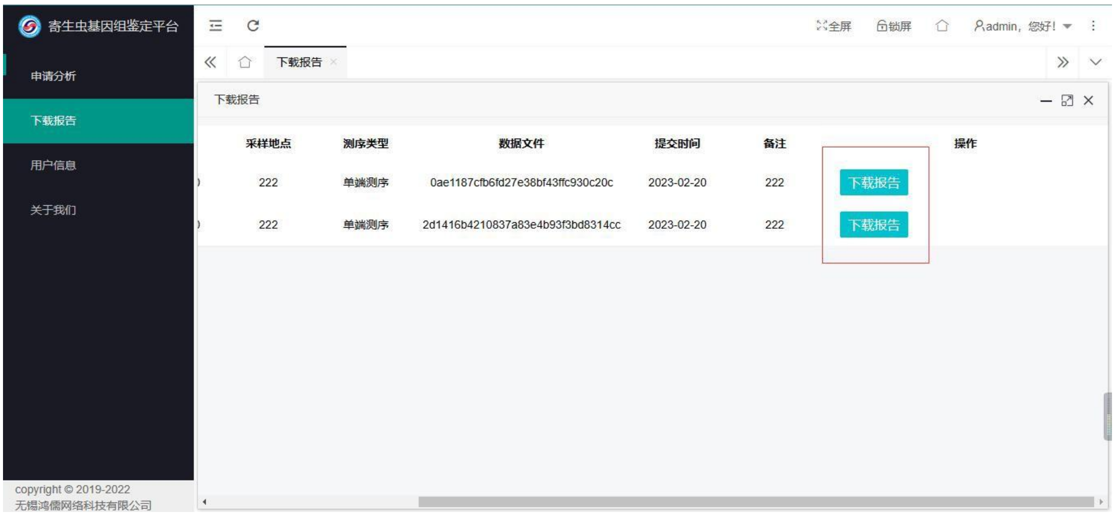

6 User Information Management

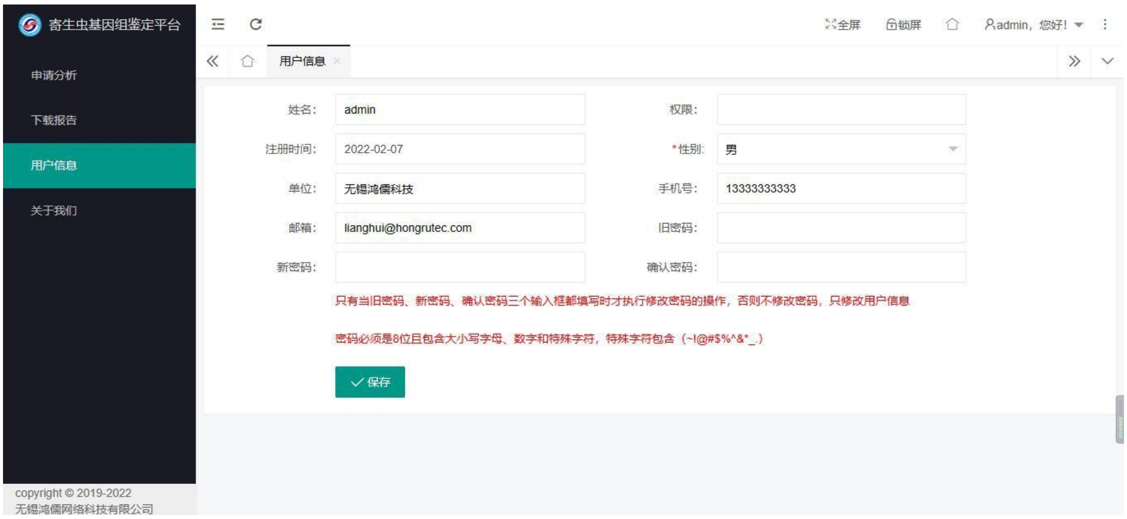

The User Profile Interface is designed for managing account information of the currently logged-in user, including functionalities such as password modification and username updates.

To modify the password, all three fields (current password, new password, and confirmation password) must be populated. The password change operation will only be executed when:

(1) The "current password" matches the active login credential.

(2) The "new password" and "confirmation password" fields contain identical strings.

Note: Passwords must meet the following complexity criteria:

- Minimum 8-character length.
- Inclusion of uppercase letters, lowercase letters, numerals, and special characters (~!@#$%^&*_.).
