# Supplementary material for: PGIP: a web server for the rapid taxonomic identification of parasite genomes
Source: Parasit Vectors. 2025 Aug 28;18:365. doi: 10.1186/s13071-025-07007-3 (PMC12392538; doi:10.1186/s13071-025-07007-3)
Supplement: Supplementary file 4 — Additional file 4: File.S4. The platform report of test sequencing data. [file 13071_2025_7007_MOESM4_ESM.pdf]

The platform report of test sequencing data

Sample 1

Results

Rank	Latin Name	Mapped reads	Relative Abundance (%)
Species	*Plasmodium vivax*	424,506	81.26
Species	*Spirometra erinaceieuropaei*	22,424	4.29
Species	*Plasmodium ovale*	19,862	3.80
Species	*Toxoplasma gondii*	7649	1.46
Species	*Wuchereria bancrofti*	7078	1.35
Species	*Pristionchus exspectatus*	6603	1.26
Species	*Elaeophora elaphi*	5057	0.97
Species	*Schmidtea mediterranea*	4387	0.84
Species	*Onchocerca volvulus*	3487	0.67
Species	*Echinococcus canadensis*	3162	0.61

Quality Control Results:

Total reads	Total bases	Q20	Q30	GC Content
51,353,148	5,027,103,999	98.69%	94.04%	42.67%

Sample 2

Results

Rank	Latin Name	Mapped reads	Relative Abundance (%)
Species	*Entamoeba histolytica*	7	70.00
Species	*Globodera rostochiensis*	1	10.00
Species	*Entamoeba dispar*	1	10.00
Species	*Entamoeba invadens*	1	10.00
Species	*Hemiselmis andersenii*	0	0.00
Species	*Cryptomonas paramecium*	0	0.00
Species	*Guillardia theta*	0	0.00
Species	*Macrostomum lignano*	0	0.00
Species	*Schmidtea mediterranea*	0	0.00
Species	*Protopolystoma xenopodis*	0	0.00

Quality Control Results:

Total reads	Total bases	Q20	Q30	GC Content
1520	50805	73.17%	37.70%	30.38%

Sample 3

Results

Rank	Latin Name	Mapped reads	Relative Abundance (%)
Species	*Angiostrongylus cantonensis*	26,143,152	70.96
Species	*Angiostrongylus costaricensis*	1,455,060	3.95
Species	*Caenorhabditis japonica*	51,480	0.14
Species	*Nippostrongylus brasiliensis*	36,286	0.10
Species	*Haemonchus contortus*	31,759	0.09
Species	*Toxocara canis*	29,234	0.08
Species	*Heligmosomoides polygyrus*	28,353	0.08
Species	*Caenorhabditis waitukubuli*	26,312	0.07
Species	*Pristionchus mayeri*	26,015	0.07
Species	*Bursaphelenchus xylophilus*	20,266	0.06

Quality Control Results:

Total reads	Total bases	Q20	Q30	GC Content
73,690,202	7,230,332,133	99.01%	95.42%	39.99%

Sample 4

Results

Rank	Latin Name	Mapped reads	Relative Abundance (%)
Species	*Clonorchis sinensis*	84,209,848	95.15
Species	*Opisthorchis felineus*	202,150	0.23
Species	*Opisthorchis viverrini*	196,343	0.22
Species	*Caenorhabditis tribulationis*	10,807	0.01
Species	*Echinostoma caproni*	4925	0.01
Species	*Caenorhabditis brenneri*	9022	0.01
Species	*Schistosoma japonicum*	4747	0.01
Species	*Cavenderia fasciculata*	229	0.00
Species	*Plasmodium cynomolgi*	39	0.00
Species	*Plasmodium relictum*	10	0.00

Quality Control Results:

Total reads	Total bases	Q20	Q30	GC Content
177,024,540	16,770,966,599	98.54%	92.12%	43.80%

Sample 5

Results

Rank	Latin Name	Mapped reads	Relative Abundance (%)
Species	*Ascaris lumbricoides*	14,093,901	77.50
Species	*Ascaris suum*	3,397,933	0.12
Species	*Ditylenchus dipsaci*	145,329	0.11
Species	*Anisakis simplex*	69,859	0.04
Species	*Caenorhabditis sulstoni*	59,841	0.04
Species	*Toxocara canis*	45,532	0.04
Species	*Angiostrongylus cantonensis*	24,883	0.03
Species	*Steinernema carpocapsae*	23,743	0.02
Species	*Oesophagostomum dentatum*	16,587	0.02
Species	*Parascaris univalens*	16,512	0.02

Quality Control Results:

Total reads	Total bases	Q20	Q30	GC Content
107,342,836	13,188,207,733	97.56%	94.96%	33.60%

Sample 6

Results

Rank	Latin Name	Mapped reads	Relative Abundance (%)
Species	*Schistosoma japonicum*	4,035,842	90.69
Species	*Trichinella nativa*	9370	0.21
Species	*Trichinella sp. T9*	5429	0.12
Species	*Trichinella pseudospiralis*	4953	0.11
Species	*Trichobilharzia regenti*	1145	0.03
Species	*Trichinella britovi*	1149	0.03
Species	*Trichinella zimbabwensis*	668	0.02
Species	*Echinostoma caproni*	750	0.02
Species	*Wuchereria bancrofti*	823	0.02
Species	*Schistosoma margrebowiei*	668	0.02

Quality Control Results:

Total reads	Total bases	Q20	Q30	GC Content
8,929,720	1,079,578,834	97.99%	95.73%	36.21%

Sample 7

Results

Rank	Latin Name	Mapped reads	Relative Abundance (%)
Species	*Schistosoma haematobium*	5,604,571	76.82
Species	*Schistosoma mattheei*	446,683	6.12
Species	*Schistosoma curassoni*	433,630	5.94
Species	*Schistosoma bovis*	367,554	5.04
Species	*Schistosoma margrebowiei*	246,671	3.38
Species	*Clonorchis sinensis*	33,032	0.45
Species	*Schistosoma mansoni*	30,046	0.41
Species	*Schistosoma rodhaini*	23,666	0.32
Species	*Schistosoma japonicum*	14,093	0.19
Species	*Opisthorchis viverrini*	12,107	0.17

Quality Control Results:

Total reads	Total bases	Q20	Q30	GC Content
24,803,138	3,097,410,634	93.82%	89.05%	37.20%

Sample 8

Results

Rank	Latin Name	Mapped reads	Relative Abundance (%)
Species	*Enterobius vermicularis*	408,194	92.65
Species	*Panagrolaimus superbus*	8220	1.87
Species	*Caenorhabditis angaria*	7523	1.71
Species	*Trichuris trichiura*	3176	0.72
Species	*Hydatigera taeniaeformis*	1144	0.26
Species	*Parascaris univalens*	521	0.12
Species	*Cylicostephanus goldi*	446	0.10
Species	*Acrobeloides nanus*	425	0.10
Species	*Gongylonema pulchrum*	402	0.09
Species	*Macrostomum lignano*	376	0.09

Quality Control Results:

Total reads	Total bases	Q20	Q30	GC Content
1,048,780	104,605,020	98.35%	93.66%	34.15%

Sample 9

Results

Rank	Latin Name	Mapped reads	Relative Abundance (%)
Species	*Toxoplasma gondii*	2,373,654	59.23
Species	*Dirofilaria immitis*	672,762	1.19
Species	*Spirometra erinaceieuropaei*	40,609	1.09
Species	*Fasciola hepatica*	26,970	0.46
Species	*Parascaris equorum*	24,885	0.17
Species	*Soboliphyme baturini*	22,576	0.08
Species	*Schistosoma japonicum*	25,090	0.06
Species	*Hammondia hammondi*	24,760	0.06
Species	*Onchocerca ochengi*	21,742	0.06
Species	*Trichobilharzia regenti*	24,354	0.05

Quality Control Results:

Total reads	Total bases	Q20	Q30	GC Content
92,256,308	13,800,370,851	100.00%	100.00%	45.18%

Sample 10

Results

Rank	Latin Name	Mapped reads	Relative Abundance (%)
Species	*Clonorchis sinensis*	22,037	46.00
Species	*Entamoeba nuttalli*	8735	18.23
Species	*Schmidtea mediterranea*	8071	16.85
Species	*Trichuris trichiura*	7885	16.46
Species	*Globodera pallida*	7366	15.38
Species	*Toxocara canis*	7074	14.77
Species	*Fasciola hepatica*	6818	14.23
Species	*Parastrongyloides trichosuri*	6331	13.22
Species	*Haemonchus placei*	5932	12.38
Species	*Syphacia muris*	5641	11.78

Quality Control Results:

Total reads	Total bases	Q20	Q30	GC Content
46,487,264	6,968,340,791	98.64%	95.32%	45.31%

Sample 11

Results

Rank	Latin Name	Mapped reads	Relative Abundance (%)
Species	*Plasmodium falciparum*	1,169,115	69.79
Species	*Spirometra erinaceieuropaei*	89,315	5.33
Species	*Plasmodium ovale*	74,382	4.44
Species	*Toxoplasma gondii*	70,892	4.23
Species	*Plasmodium reichenowi*	37,501	2.24
Species	*Pristionchus exspectatus*	17,634	1.05
Species	*Wuchereria bancrofti*	15,156	0.90
Species	*Onchocerca volvulus*	15,079	0.90
Species	*Schmidtea mediterranea*	10,782	0.64
Species	*Plasmodium gaboni*	6748	0.40

Quality Control Results:

Total reads	Total bases	Q20	Q30	GC Content
82,875,966	17,036,420,586	97.49%	90.96%	42.44%

Sample 12

Results

Rank	Latin Name	Mapped reads	Relative Abundance (%)
Species	*Naegleria fowleri*	50,410	29.40
Species	*Spirometra erinaceieuropaei*	33,917	19.78
Species	*Toxoplasma gondii*	14,393	8.39
Species	*Wuchereria bancrofti*	9785	5.71
Species	*Pristionchus exspectatus*	7805	4.55
Species	*Schmidtea mediterranea*	5644	3.29
Species	*Onchocerca volvulus*	3428	2.00
Species	*Dirofilaria immitis*	1807	1.05
Species	*Parapristionchus giblindavisi*	1793	1.05
Species	*Pristionchus pacificus*	1698	0.99

Quality Control Results:

Total reads	Total bases	Q20	Q30	GC Content
88,932,436	13,334,215,262	98.76%	95.97%	44.68%

Sample 13

Results

Rank	Latin Name	Mapped reads	Relative Abundance (%)
Species	*Spirometra erinaceieuropaei*	28,584,220	93.37
Species	*Schistocephalus solidus*	65,354	0.21
Species	*Dibothriocephalus latus*	23,176	0.08
Species	*Trichobilharzia regenti*	2965	0.01
Species	*Schistosoma margrebowiei*	2894	0.01
Species	*Fasciola hepatica*	1864	0.01
Species	*Echinostoma caproni*	1788	0.01
Species	*Schistosoma japonicum*	1614	0.01
Species	*Schmidtea mediterranea*	1583	0.01
Species	*Macrostomum lignano*	1543	0.01

Quality Control Results:

Total reads	Total bases	Q20	Q30	GC Content
61,488,424	9,174,435,098	98.15%	94.55%	43.80%

Sample 14

Results

Rank	Latin Name	Mapped reads	Relative Abundance (%)
Species	*Plasmodium falciparum*	2,632,974	78.32
Species	*Dirofilaria immitis*	6515	0.19
Species	*Caenorhabditis angaria*	6330	0.19
Species	*Trichuris trichiura*	5711	0.17
Species	*Strongylus vulgaris*	4898	0.15
Species	*Plasmodium reichenowi*	4811	0.14
Species	*Panagrolaimus superbus*	4372	0.13
Species	*Trypanosoma cruzi*	3911	0.12
Species	*Clonorchis sinensis*	2841	0.08
Species	*Meloidogyne hapla*	1261	0.04

Quality Control Results:

Total reads	Total bases	Q20	Q30	GC Content
6,727,352	983,723,166	98.56%	95.90%	37.88%

Sample 15

Results

Rank	Latin Name	Mapped reads	Relative Abundance (%)
Species	*Spirometra erinaceieuropaei*	27,119	0.85
Species	*Toxoplasma gondii*	26,862	0.84
Species	*Plasmodium ovale*	25,125	0.79
Species	*Wuchereria bancrofti*	8615	0.27
Species	*Pristionchus exspectatus*	7542	0.24
Species	*Onchocerca volvulus*	6005	0.19
Species	*Schmidtea mediterranea*	4621	0.14
Species	*Parapristionchus giblindavisi*	1722	0.05
Species	*Pristionchus pacificus*	1607	0.05
Species	*Fasciola gigantica*	1128	0.04

Quality Control Results:

Total reads	Total bases	Q20	Q30	GC Content
67,928,034	10,163,879,458	98.00%	94.25%	40.92%
