# Supplementary material for: PGIP: a web server for the rapid taxonomic identification of parasite genomes
Source: Parasit Vectors. 2025 Aug 28;18:365. doi: 10.1186/s13071-025-07007-3 (PMC12392538; doi:10.1186/s13071-025-07007-3)
Supplement: Supplementary file 5 — Additional file 5: File.S5. PGIP report template. [file 13071_2025_7007_MOESM5_ESM.pdf]

Genome sequencing of predicted parasite sample

Name: -	Sample ID：sample23111001	Submitting Institution: Outpatient Department
Gender: Male	Sample Type: Parasitic tissue	Clinical Diagnosis: Suspected sparganum infection
Age: 46	Date：2023-11-10	

Testing item: Parasite identification

Detection Method: Metagenomic Sequencing

Results:

Rank	Latin Name	Mapped reads	Relative Abundance (%)
1	*Spirometra erinaceieuropaei*	28,584,220	93.37
2	*Schistocephalus solidus*	65,354	0.21
3	*Dibothriocephalus latus*	23,176	0.08
4	*Trichobilharzia regenti*	2,965	0.01
5	*Fasciola hepatica*	1,864	0.01
6	*Schistosoma japonicum*	1,614	0.01
7	*Schistosoma margrebowiei*	2,894	0.01
8	*Macrostomum lignano*	1,543	0.01
9	*Schmidtea mediterranea*	1,583	0.01
10	*Echinostoma caproni*	1,788	0.01

Quality Control Results:

Total reads	Total bases	Q20	Q30	GC Content
61488424	9174435098	98.15%	94.55%	43.80%

Note: Q20/Q30 represent the error probability assigned during base calling in sequencing. Q20 indicates a 1% error rate (99% accuracy), while Q30 indicates a 0.1% error rate (99.9% accuracy). The minimum acceptable thresholds are typically ≥90% for Q20 and ≥85% for Q30.

Conclusion: Sparganum infection.

Disclaimer

1. This assay is a non-routine clinical test intended for reference purposes only. Clinical interpretation must be conducted by licensed physicians. Any objections must be raised within seven work days.
2. Improper procedures in specimen collection, preservation, or transportation may compromise the analytical performance of this assay.
3. Results are valid solely for the submitted specimen. Methodological detection limits, subthreshold pathogen load, database limitations, or prior medication may result in undetected pathogens.
4. The testing provider shall rigorously protect patient privacy and test results in accordance with applicable laws.

Performed by:　　　　Reviewed by:　　　　Report Date: 2023-11-17

Address: 117 Yangxiang, Meiyuan, Wuxi City, Jiangsu Province, China
Postal Code: 214064　　Email: src@jipd.com
